# Ty retrotransposon element based multiple integration toolkit for *Saccharomyces cerevisiae*

**DOI:** 10.1016/j.synbio.2025.04.011

**Published:** 2025-04-23

**Authors:** Song Gao, Weizhu Zeng, Dong Li, Sha Xu, Jingwen Zhou

**Affiliations:** a Engineering Research Center of Ministry of Education on Food Synthetic Biotechnology, Jiangnan University, 1800 Lihu Road, Wuxi, Jiangsu, 214122, China; b School of Food Science and Technology, Jiangnan University, 1800 Lihu Road, Wuxi, Jiangsu, 214122, China; c Science Center for Future Foods, Jiangnan University, 1800 Lihu Road, Wuxi, Jiangsu, 214122, China; d Key Laboratory of Industrial Biotechnology, Ministry of Education and School of Biotechnology, Jiangnan University, 1800 Lihu Road, Wuxi, Jiangsu, 214122, China

**Keywords:** Retrotransposons, High copy number integration, Gene overexpression, Metabolic engineering, Protein production

## Abstract

Extra-high-level overexpression of single or multiple specific proteins by integrating specific genes in the genome is vital to achieve the stable and efficient production of target proteins and metabolites in *S. cerevisiae*. Five families of Ty elements in the genome of *S. cerevisiae* CEN.PK2-1D, which could have dozens to hundreds of copies, have been employed to achieve massive gene expression. By engineering nine selective markers, six of them (*TRP1*, *LEU2*, *URA3*, *HIS5*, *natMX* and *hphMX*) achieve stably high copy integration (>15 copies) at Ty sites. Fluorescence proteins and taxifolin biosynthesis pathway genes were overexpressed to verify the toolkit. The titer of protein phiYFP in the multiple integration strain reached 1.6 g/L (268.1 mg/g DCW), and its fluorescence intensity was 3.3 times higher than that in the episomal overexpression strain. For taxifolin biosynthesis, 14 genes were integrated into three different Ty sites using three selective markers from the toolkit, resulting in 277.6 mg/L taxifolin accumulation from glucose.

## 1 Introduction

Gene overexpression is a common way to improve protein or chemical compound accumulation in *Saccharomyces cerevisiae* [1,2]. Episomal expression in plasmids can achieve a high copy number of heterologous genes and is widely used in both construction and screening. The instability of episomal expression limits its application on an industrial scale. Therefore, researchers have developed a auxotrophic marker expression cassette [3] and degradation signal [4] to improve the stability of the plasmids under high copy numbers. However, it is still unstable and could bring an additional metabolic burden to the host, especially when transforming multiple plasmids in a single cell. Homologous recombination-based genome integration is a common method used to achieve the expression of genes in *S. cerevisiae* because it is highly stable and results in a limited metabolic burden. The most obvious disadvantage of this method is that single-site integration can only achieve a very low expression level, and thus could not achieve high efficiency in the accumulation of proteins or other target compounds [5].

To address the limitations of the episomal and single-site integration expression methods, the multiple integration expression of genes in the *S. cerevisiae* genome is an ideal choice. The most used multiple integration sites in *S. cerevisiae* are rDNA and δ sites. A typical rDNA site is a 100–200 repeat sequence in the yeast genome and each repeat is ∼9.1 kb in length [6]. However, rDNA sites are sequences that are tandemly repeated on Chromosome XII, making multiple integration strains unstable because they could homologously recombine with each other. The δ site is a type of Ty element, which includes Ty1Cons1, Ty1Cons2 and Ty2Cons, and could be developed for multiple gene integration sites in *S. cerevisiae* [7]. In addition to the δ site, σ, τ and ω sites, which are known as Ty3Cons, Ty4Cons and Ty5Cons [8], also exist in the *S. cerevisiae* genome. The δ site has more than 300 copies in the genome of *S. cerevisiae* CEN.PK2-1D, whereas σ and τ sites have 30–40 copies. This suggests that σ and τ sites could also be useful in achieving multiple integration in *S. cerevisiae* [9].

The Ty retrotransposon is a ∼6 kb transposon with a high copy number. “Ty” means retrotransposon (T) elements existing in yeast (y). Ty elements have been studied for more than 40 years and are known to be related to yeast mating [10], chromosome deletions/duplications [11] and specific phenotypes of *S. cerevisiae*. For example, the auxotrophic phenotype *URA3-52* is caused by a 6.1 kb Ty52 insertion at the *URA3* site [12]. Ty elements are flanked by long terminal repeats (LTRs) or solo LTR sequences of 251–371 bp in length [13,14]. The distribution of these Ty elements in the genome of *S. cerevisiae* is far more random than that of rDNA. The LTR sequences can be used as homology arms of homologous recombination. The Ty elements constitute about 0.04 % of the total DNA. However, Ty-related mRNA constitutes about 5–10 % of the total mRNA [15], suggesting that the Ty sites are ideal for the multiple integration of genes and efficient overexpression. Ty retrotransposon element based multiple integration has been previously developed for gene overexpression [9,16]. The selective marker *KlURA3* with a degradation signal has an excellent performance in high integration copy numbers, however, other auxotrophic and resistance selective markers are not as well developed.

To achieve a very high level of protein expression in *S. cerevisiae*, four auxotrophic genes *ScTRP1*, *KlLEU2*, *KlURA3* and *SpHIS5* and five resistance genes *natMX*, *hphMX*, *kanMX*, *patMX* and *bleMX* were chosen as selective markers. Ty1cons1, Ty1Cons2, Ty2Cons, Ty3Cons and Ty4Cons sites were chosen as the multiple integration sites. Degradation signal CL-1 [4], weak promoters and non-AUG initiation codons were chosen to down-regulate selective gene expression. A multiple integration toolkit containing 55 plasmids was built. To verify the ability of the toolkit to integrate at multiple Ty sites at the same time, taxifolin biosynthesis pathway genes were integrated into three different Ty sites with three selective markers. The integration size of the DNA fragment length could be as large as 8.4 kb, and the integration copy numbers could be as high as 16. A total of four fluorescence proteins EGFP, phiYFP, mKate2 and mKOk were integrated at four different Ty sites under four selective markers. The concentration of the phiYFP protein was 1.6 g/L, accounting for 48.8 % of the total protein accumulation. The fluorescence intensity and taxifolin production of the multiple integration strains did not significantly reduce even after 15 generations. This confirmed that the Ty-based multiple integration toolkit described here is fit for gene overexpression with high integration of copy numbers.

## 2 Materials and methods

### 2.1 Strains, plasmids, genes and chemicals

*S. cerevisiae* CEN.PK2-1D (*MATα*, *ura3-52*, *trp1-289*, *leu2-3,112*, *his3Δ1*, *MAL2-8*^*c*^, *SUC2*) [17] was used for gene integration. *Escherichia coli* JM109 was used for plasmid maintenance and propagation. The T-vector pMD19T-simple (TaKaRa, Dalian, China) was used for gene construction. Plasmids pcfB2989, pcfB2988, pcfB2797, pcfB2990, pcfB2796 and pcfB2803 were gifts from Irina Borodina and Jerome Maury (Addgene plasmid #63636, #63638, #63639, #63645, #63641, #63646) [9]. The plasmids contain the LTR sequences of Ty1cons1, Ty1Cons2, Ty2Cons, Ty3Cons and Ty4Cons. Plasmids pcfB2989, pcfB2803, pUC57-TRP1-anti, pUG27, pUG6, pUG66, pAG25, pAG31 and pAG32, which contain *KlURA3*, *KlLEU2*, *ScTRP1*^anti^, *SpHIS5*, *KanMX*, *bleMX*, *natMX*, *patMX* and *hphMX*, were used for the PCR amplification of selective markers and degradation signal CL-1(deg) [18,19]. The genes EGFP, phiYFP [20], mKOk [21], and mKate2 [22] were synthesized by Sangon (Shanghai, China). Hygromycin B solution, G418 sulfate and bleomycin were purchased from Sangon (Shanghai, China). Nourseothricin sulfate was purchased from Solarbio (Beijing, China). Ammonium glufosinate was purchased from Ark Pharm (Libertyville, IL, USA). The mother solutions of nourseothricin sulfate, hygromycin B, ammonium glufosinate, G418 sulfate and bleomycin were 100, 300, 400, 200 and 100 g/L, while the working concentration was 100, 300, 800, 200 and 100 mg/L, respectively. A ClonExpress Ultra One Step Cloning Kit was used for Gibson assembly and purchased from Vazyme (Nanjing, Jiangsu, China). Yeast nitrogen base (YNB, without amino acids and ammonium sulfate) medium was purchased from Sangon (Shanghai, China). Chemicals not mentioned were all purchased from Sinopharm Chemical Reagent Co., Ltd. (Shanghai, China).

**Fig. 1:**
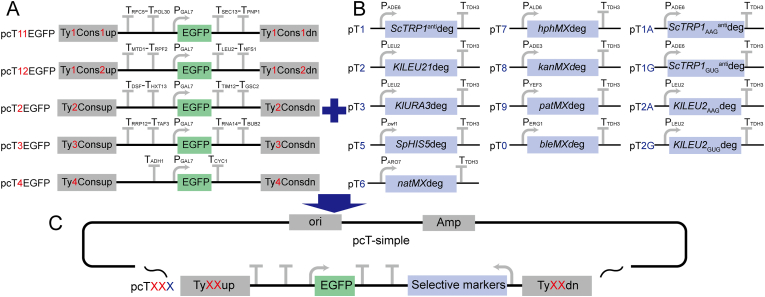
Characteristics of the multiple integration toolkit. A: The characteristics of the integration plasmid skeletons without selective markers. B: The characteristics of selective markers. C: The characteristics of the multiple integration plasmid used. The Xs indicate the plasmid number.

Plasmid genotypes are listed in Table S1. Strain genotypes are listed in Table S2. The key genes, promoters, terminators and LTR sequences are listed in Table S3. All primers are listed in Table S4. The qPCR data are listed in Tables S5–S8. The main features of the plasmids are described in Fig. 1.

### 2.2 Yeast transformation and detection of fluorescence intensity

All integration expression frames were amplified by primer pairs from plasmids(Ty11-inte-F/R, Ty12-inte-F/R, Ty2-inte-F/R, Ty3-inte-F/R, Ty4-inte-F/R and rDNA-F/R. The PCR products were purified with the phenol-chloroform method [23] and diluted to ∼500 ng/μL. Yeast cells were transformed by a lithium acetate method [24]. When the selective markers were *TRP1*, *LEU2*, *URA3*, *HIS5* or *patMX*, the transformants were spread on YNB agar plates. When the selective markers were *natMX*, *hphMX*, *kanMX* or *bleMX*, the transformants were suspended in 4 mL YPD, cultivated at 30 °C, 220 rpm for 8–12 h, then resuspended in 4 mL ddH_2_O and spread on YPD agar plates. The plates were incubated at 30 °C for 5–7 days until colonies were visible.

### 2.3 Cultivation conditions

After integration, the recombinant strains were randomly picked and transferred onto 48 deep well plates with 1.5 mL YNB medium in each well. The plates were incubated at 30 ^o^C with 220 rpm for 18–20 h. The culture was used for cell density, fluorescence intensity and RT-qPCR measurement.

For the protein accumulation assay, the strains C800E, C800Y, C800O and C800K, were cultivated in a 250 mL shake flask with 10 mL YNB medium at 30 °C with 220 rpm for 24 h. The strains C811E, C812E, C802Y, C803O and C804K were cultivated in a 250 mL shake flask with 50 mL YPD medium at 30 °C with 220 rpm for 24 h. The cultures were used for protein extraction, SDS-PAGE (SDS-polyacrylamide gel electrophoresis), cell density, fluorescence intensity and RT-qPCR.

For the (2*S*)-taxifolin biosynthesis assay in 48-deep-well plates, the recombinant strains were randomly picked and added to 48-deep-well plates with 1.5 mL YPD medium in each well and cultured at 30 °C with 220 rpm for 48 h. The YPD medium was supplemented with 1 % (*V*/*V*) ethanol, 500 mg/L *p*-coumaric acid or naringenin depending on gene integration conditions. After HPLC analysis, the best-performing colonies were selected for shake flask cultivation.

For the (2*S*)-taxifolin biosynthesis assay in shake flasks, the recombinant strains were cultivated in 10 mL YPD medium in a 250-mL shake flask at 30 °C with 220 rpm for 14–16 h. Then, 20 mL of YPD medium was inoculated with 1 % (*V*/*V*) cultures in a 250-mL shake flask at 30 °C with 220 rpm for 72 h. At 12, 24, 36 and 48 h, 0.5 % (*V*/*V*) 500 g/L glucose (for strains C8011, Y621, C901 and Y732), 0.5 % (*V*/*V*) 50 g/L *p*-coumaric acid in 100 % ethanol (for strains C803 and C857) or 0.5 % (*V*/*V*) 50 g/L naringenin in 100 % ethanol (for strains C805 and Y543) were added to the medium.

For strain Y732 cultivation in 5-L bioreactor, the seed culture was prepared the same as described above. Then, 10 % (*V*/*V*) of the seed culture was reinoculated into 2.5 L of YPD medium. The fermentation conditions were 30 °C at 400 rpm and a 2.5 vvm (volume of air per volume) airflow rate. At 18 h, start feeding 500 g/L glucose under 10 mL/h. The pH was maintained at 5.5 ± 0.1 by adding 4 M NaOH, while the dissolved oxygen (DO) was maintained at 40 ± 5 % by controlling the agitation between 400 and 800 rpm.

For protein phiYFP extraction, the strain C802Y was cultivated in a 250 mL shake flask containing 10 mL YPD at 30 °C with 220 rpm for 16 h. Then, 2 % (*V*/*V*) culture was reinjected into a 250 mL shake flask containing 50 mL YPD and cultured at 30 °C with 220 rpm for 24 h.

Amino acids (50 mg/L) were added to the YNB medium if necessary.

### 2.4 Fluorescence detection

The excitation wavelength/emission wavelength of EGFP, phiYFP, mKOk and mKate2 were 488/520, 525/551, 551/577 and 588/635 nm, respectively. Fluorescence intensity was detected by a microplate reader (BioTek, SYNERGY H1, Palo Alto, CA, USA). The cultivated medium was diluted 100 times with ddH_2_O, and 50000 cells were analyzed by using a fluorescence-activated cell sorting (FACS, Beckman Coulter, MoFlo XDP, Brea, CA, USA) system [25].

### 2.5 RT-qPCR assays

Yeast total DNA was extracted by a rapid DNA preparation method [26]. Using total DNA as the template, RT-qPCR was conducted using ChamQ Universal SYBR qPCR Master Mix (Vazyme, Nanjing, Jiangsu, China) on a lightCycler 480II thermal cycler system (Roche, Mannheim, Germany). The housekeeping gene *ACT1* was used as the reference gene. The strain C877 (has one copy of EGFP in the genome), Y200 (has one copy of CHS and *aroL* in the genome) and C100 (has one copy of F3′H in the genome) was used as reference strain. The gene copy number was represented by the value of -ΔΔC_T_, and the fold change of the genes was represented by the value of 2^-ΔΔC_T_ [27]. The primers used in the RT-qPCR are described in Table S4.

### 2.6 High-pressure homogenization

After cultivation, the strain C802Y were washed twice with phosphate buffer solution (PBS, pH 7.4) and resuspended in PBS (pH 7.4) to an OD_600_ of 25. The cells were then lysed by high-pressure homogenization [28]. The high-pressure homogenization was conducted at 100 MPa and repeated for six cycles.

### 2.7 SDS-PAGE analysis

The strains were washed twice with PBS (pH 7.4) and resuspended by urea (8 M) to an OD_600_ of 5. Then, 100 μL of 0.5 mm acid-treated glass beads were mixed with 500 μL of suspension. The mixture was lysed by FastPrep-24™ 5G (M.P. Biomedicals, Santa Ana, CA, USA) under the recommended program (6 m/s) for six cycles, and then centrifuged at 10,000×*g* for 5 min. The supernatant was mixed with SDS-PAGE loading buffer (4×) and heated in a boiling water bath for 10 min. 10 μL of the mixture was used for SDS-PAGE analysis.

### 2.8 Protein concentration analysis

After high-pressure homogenization, the suspension was centrifuged at 10,000×*g* for 20 min at 4 °C. The supernatant was used for protein concentration analysis. Total protein concentration was detected by using a BCA Protein Assay kit (Sangon, Shanghai, China). The suspension was diluted 10 times and used for SDS-PAGE analysis. After SDS-PAGE analysis, the concentration of phiYFP was calculated based on its proportion.

### 2.9 HPLC analysis

The fermented medium was diluted 2–10 times (*V*/*V*) with methyl alcohol and mixed well. The mixture was centrifuged at 12,000×*g* for 5 min and the supernatant was filtered by using a 0.22 μm nylon membrane. The HPLC method was as described before [29].

## 3 Results

### 3.1 Characteristics of the multiple integration toolkit

The five Ty element LTR sequences (Ty1Cons1, Ty1Cons2, Ty2Cons, Ty3Cons and Ty4Cons), nine selective markers (*ScTRP1*^anti^deg, *KlLEU2*deg, *KlURA3*deg, *SpHIS5*deg, *natMX*deg, *hphMX*deg, *KanMX*deg, *patMX*deg and *bleMX*deg) and weak terminators were assembled using Gibson assembly to yield the multiple integration toolkit. The genotype of the original strain, *trp1-289*, is attributed to a single base mutation, C403T (CAG to UAG; resulting in a premature stop codon). Therefore, the DNA sequence of *ScTRP1*deg was then optimized based on *Escherichia coli*'s codon preference to generate *ScTRP1*^anti^deg, reducing the potential for homologous recombination repair of the *trp1-289* genome by *ScTRP1*^anti^deg. To reduce expression levels of *ScTRP1*^anti^deg and *KlLEU2*deg, the initial coding sequence was replaced by AAG or GUG, resulting in *KlLEU2*_AAG_deg, *ScTRP1*_AAG_^anti^deg, *KlLEU2*_GUG_deg and *ScTRP1*_GUG_^anti^deg (Fig. 1). The plasmids in each group feature unique LTRs and terminators, which could alleviate instability caused by homologous recombination when choosing more than two Ty sites for multiple integration.

### 3.2 Integration ability of the multiple integration toolkit

**Fig. 2:**
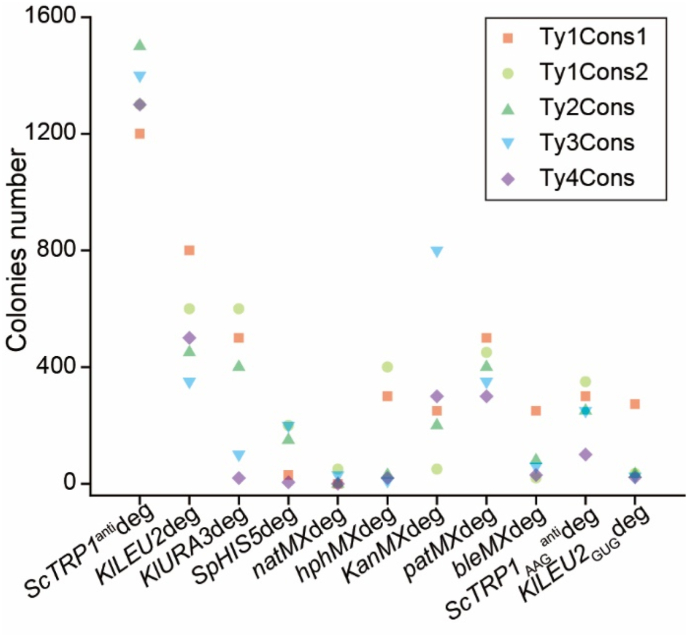
Distribution of colony numbers after a single transformation. Different selective markers have different colony distributions after a single transformation.

**Fig. 3:**
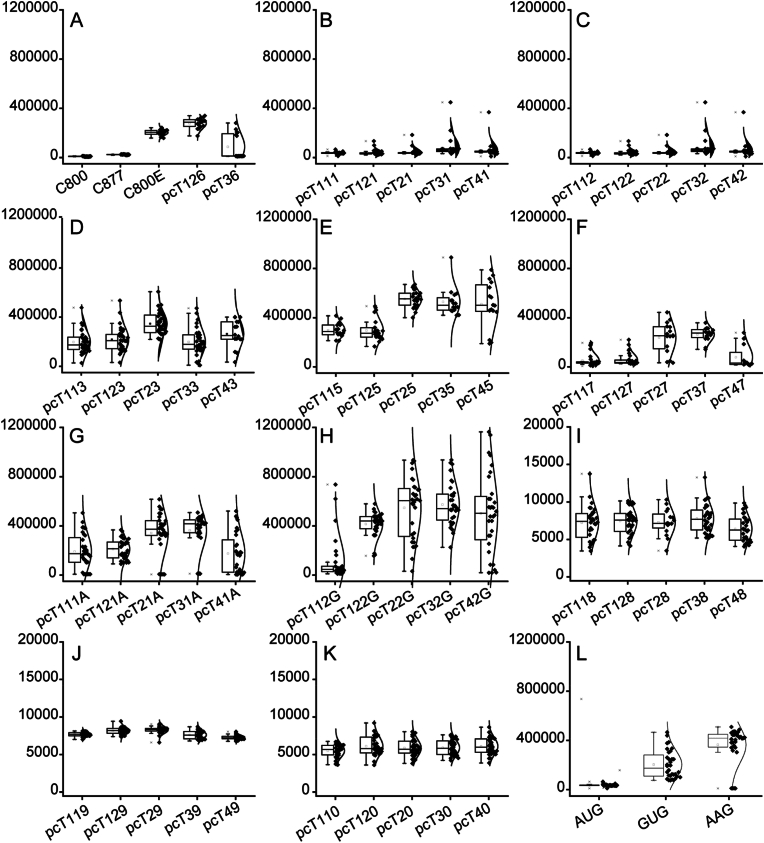
Distribution of the fluorescence intensity of colonies. The Y-axis is fluorescence intensity (The cell density of OD_600_ was up to 5.0). A: C800 is the initial strain without EGFP. C877 has one copy of EGFP in the genome. Strain C800E is C800 with the plasmid pY26-P_GAL7_-EGFP. The pcT126 and pcT36 are the EGFP expression boxes that were integrated into the C800 genome at Ty1Cons2 and Ty3Cons sites under *natMXdeg* selective markers. B–K: Distribution of fluorescence intensity in colonies with five Ty sites under selective markers *ScTRP1*^anti^deg, *KlLEU2*deg, *KlURA3*deg, *SpHIS5*deg, *hphMX*deg, *ScTRP1*_AAG_^anti^deg, *KlLEU2*_GUG_deg, *KanMX*deg, *patMX*deg and *bleMX*deg. L: Distribution of fluorescence intensity in colonies when selective marker *ScTRP1*^anti^deg was under the initial codons AUG, GUG and AAG.

After transformation, the colony numbers were counted and the results are shown in Fig. 2. Among the nine selective markers, *KlURA3*deg, *SpHIS5*deg and *hphMX*deg could stably result in colonies with a high intensity of fluorescence, whereas *kanMX*, *patMX* and *bleMX* cannot. Five plasmids (pcT116, pcT126, pcT26, pcT36, and pcT46) were constructed based on *natMX*deg. However, pcT116, pcT26, and pcT46 did not yield any colonies, while pcT126 and pcT36 still yielded colonies with high intensity of fluorescence (Fig. 3A). The *ScTRP1*^anti^deg and *KlLEU2*deg could obtain colonies with a high fluorescence intensity occasionally (Fig. 3B/3C). The selective markers *kanMX*, *patMX*, and *bleMX* can obtain a large number of transformants (Fig. 2), but most of the obtained transformants do not have high fluorescence intensity (Fig. 3I/3J/3K).

Because only a few transformants exhibited high intensity of fluorescence when using *ScTRP1*^anti^deg and *KlLEU2*deg as the selective markers, non-AUG initiation codons optimized *ScTRP1*^anti^deg and *KlLEU2*deg were constructed. The *ScTRP1*_AAG_^anti^deg showed better performance than *ScTRP1*_GUG_^anti^deg, whereas both are better than the initial *ScTRP1*^anti^deg (Fig. 3L). The *KlLEU2*_GUG_deg showed better performance than the initial *KlLEU2*deg, whereas the *KlLEU2*_AAG_deg did not result in any colonies (Fig. 3H). Two more selective markers (*ScTRP1*_AAG_^anti^deg and *KlLEU2*_GUG_deg) could stably result in colonies with high intensity of fluorescence after modification. Finally, a Ty site-based multiple integration toolkit was constructed with a total of 55 plasmids.

**Fig. 4:**
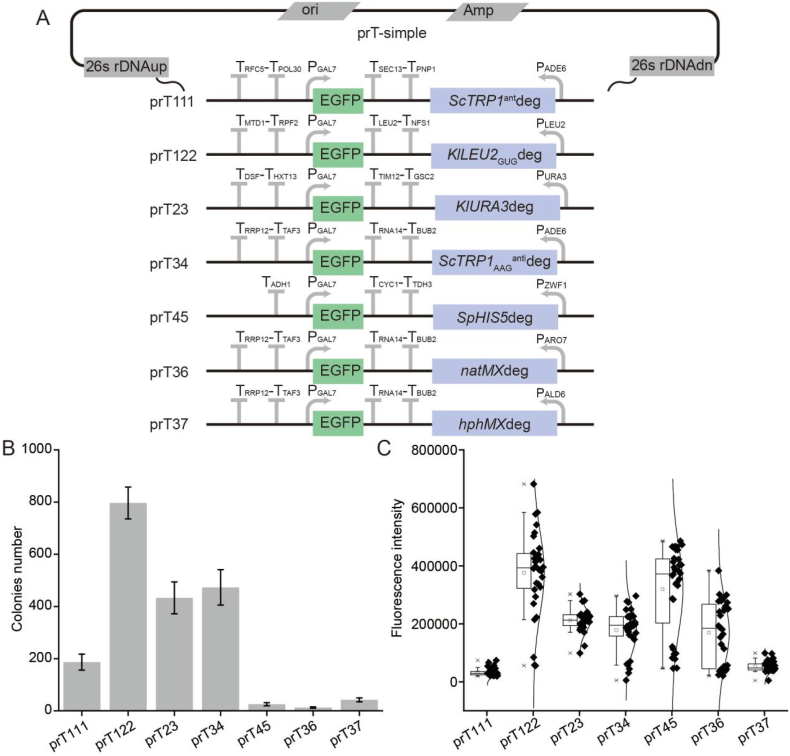
Application at the 26s rDNA site. A: The characteristics of plasmid skeletons with selective markers at 26s rDNA sites. B: The distribution of colony numbers after a single transformation. C: The fluorescence intensity distribution of the colonies.

Seven selective markers (*ScTRP1*^anti^deg, *KlLEU2*_GUG_deg, *KlURA3*deg, *ScTRP1*_AAG_^anti^deg. *SpHIS5deg*, *natMX*deg and *hphMX*deg) were also verified at 26s rDNA sites. Among them, five selective markers showed good multiple integration ability, except for *ScTRP1*^anti^deg and *hphMX* (Fig. 4). When using *KlLEU2*_GUG_deg, the transformation efficiency at the 26s rDNA site is higher than at the Ty site(number of transformants: ∼800 vs ∼300). When using *ScTRP1*^anti^deg, *KlURA3*deg, *ScTRP1*_AAG_^anti^deg. *SpHIS5deg*, *natMX*deg and *hphMX*deg, the transformation efficiency at the 26s rDNA site is similar to that at the Ty site. The fluorescence distribution of the transformants using the five selective markers at the 26s rDNA site and Ty site is largely similar.

### 3.3 The copy number distribution of integrated strains

**Fig. 5:**
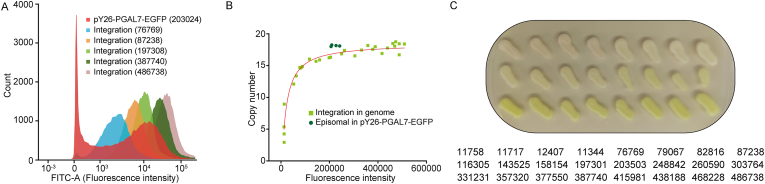
The relationship between fluorescence intensity and copy numbers. A: Distribution of the fluorescence intensity of integration strains and episomal strains analyzed by FACS. B: Distribution of the copy number of integration strains and episomal strains under different fluorescence intensity. The fluorescence intensity numbers are calculated by using a microplate reader (under 48-deep-well plate cultivation). C: The colour of integration colonies. The numbers beside the colonies are fluorescence intensity values calculated by a microplate reader (under 48-deep-well plate cultivation).

The relationship between fluorescence intensity and gene copy number was verified by RT-qPCR. The FACS results showed that the episomal strains had two peaks, which could be caused by the unstable maintenance of the episomal plasmids in the cells. The integration strains display a single peak, validating consistent expression levels among all colonies and demonstrating strong robustness (Fig. 5A). The copy number of episomal strain is approximately 19, and the fluorescence intensity is about 200,000. When the integration strains reach the 200,000, the copy number is about 15, and when the copy reaches 19, the fluorescence intensity exceeds 500,000. It can be seen that integration strains have higher expression levels at the same copy number (Fig. 5B). If the fluorescence intensity was more than 100,000, the colonies would become visibly green (Fig. 5C). It was easy to get integration stains with fluorescence intensity far beyond 100,000 (Fig. 3). This demonstrated that the toolkit could be easily used to obtain high gene expression level. Unlike the CRISPR-assisted multi-copy integration methods, this method only requires one round of transformation to obtain multiple integrated transformants [30].

### 3.4 Applications of the toolkit in protein overexpression

**Fig. 6:**
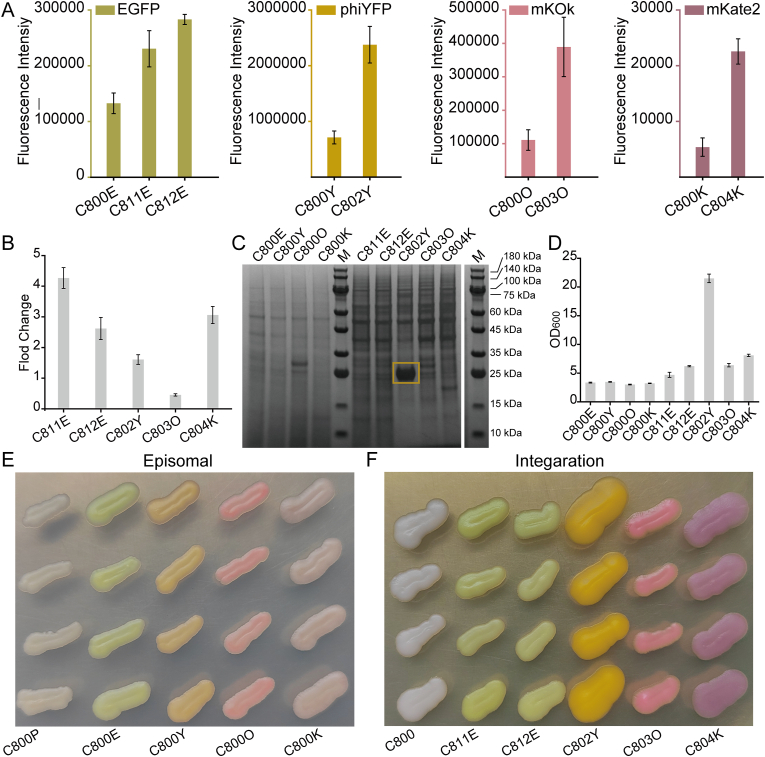
Protein overexpression assay. The fluorescence proteins EGFP, phiYFP, mKOk and mKate2 were overexpressed by using the multiple integration toolkit. A: The fluorescence intensity of episomal strains and integration strains (under shake flask cultivation). B: The fold change of genes in integration strains compared with episomal strains. C: The SDS-PAGE of episomal strains and integration strains. The phiYFP protein accumulation in the integration strain is marked with a yellow box. D: The cell density of episomal strains and integration strains for fluorescence protein production. E: The phenotype of episomal strains. F: The phenotype of integration strains.

The genes encoding fluorescence proteins (EGFP, phiYFP, mKate2 and mKOk) were integrated into Ty1Cons1, Ty1Cons2, Ty2Cons, Ty3Cons and Ty4Cons sites under *KlLEU2*_GUG_deg, *KlURA3*deg, *hphMX*deg and *ScTRP1*_AAG_^anti^deg selective markers, respectively. Then, the highest fluorescence strain was obtained from eight randomly selected single colonies. The fluorescence intensity of the multiple integrated strains was 1.74, 2.13, 3.34, 4.20 and 3.51 times higher than that of the episomal expression strains, respectively (Fig. 6A). The four fluorescence protein gene copy numbers were 4.27, 2.62, 1.61, 0.45 and 3.06 times that of the episomal expression strains, respectively (Fig. 6B).

The production of the fluorescent protein in the integrated strains was not significantly improved compared to those in the episomal expression strains at the same cell density, except for phiYFP under *KlURA3*deg selective markers (Fig. 6C). The gene *KlURA3* will complement the uracil metabolism pathway, which is crucial for strain growth. When *KlURA3* was complemented, the cell density was dramatically increased (OD_600_ = 21.5) compared with other auxotrophic genes or resistance genes that were introduced (Fig. 6D). Massive overexpression of protein phiYFP was found in strain C802, which also showed the best growth conditions among all strains (Fig. 6E and F). The protein phiYFP was extracted through the high-pressure homogenization method, the total protein and the phiYFP concentration were 3.3 g/L and 1.6 g/L, respectively. The protein yield of phiYFP accounted for 48.8 % of the total protein yield (Supplementary Fig. S1). This demonstrated that the multiple integration toolkit could be used for protein overexpression in *S. cerevisiae* after the uracil metabolism pathway was complemented.

### 3.5 Applications of the toolkit in pathway construction

**Fig. 7:**
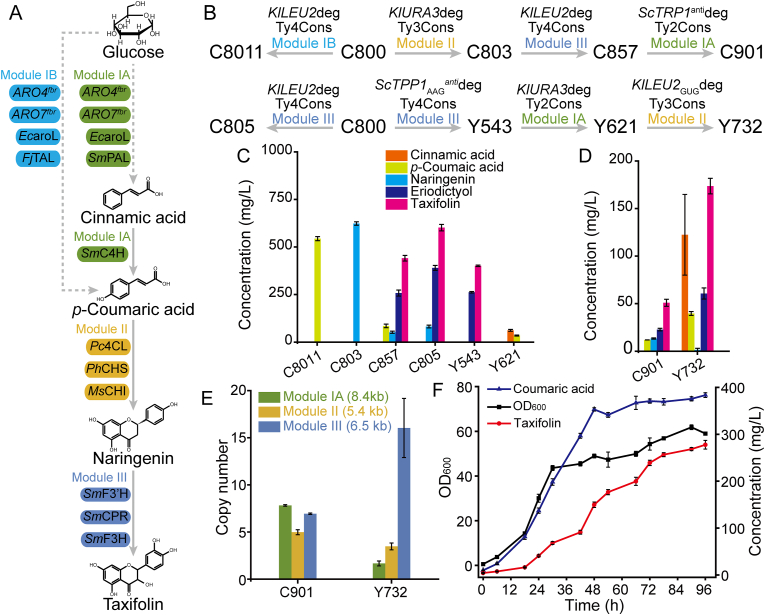
Pathway genes overexpression assay. A: Taxifolin biosynthesis pathway-related genes. B: Pathway gene integration process and strain construction process. C: Chemical production of intermediate strains. D: Chemical production of *de novo* strains. E: Gene copy numbers in *de novo* strains and the length of the expression box. F: Fed-batch fermentation of strain Y732.

The taxifolin biosynthesis pathway genes and intermediate products are shown in Fig. 7A. A series of strains were constructed and verified to find out the most important impacts affecting pathway gene integration and overexpression (Fig. 7B). The strain C8011 was integrated with Module IB genes, and 544.0 mg/L of *p*-coumaric acid was obtained from glucose without any metabolic engineering. The production was similar to previously reported engineered strains ST4048 (550 mg/L) [31] and QL05 (∼600 mg/L) [32], and much higher than a single integration strain Y200 (68.26 mg/L) [5]. Strain C803 was integrated with Module II genes, and 623.6 mg/L naringenin was obtained from 1000 mg/L *p*-coumaric acid, 1.5 times more than the episomal strain (246.6 mg/L) [25]. Strain C857 was integrated with Module II and Module III genes. The accumulation of naringenin, eriodictyol and taxifolin were 51.9, 257.8 and 441.1 mg/L from 1000 mg/L *p*-coumaric acid, and 85.2 mg/L *p*-coumaric acid was left. The strains C805 and Y543 were both integrated with Module III genes, the accumulation of eriodictyol and taxifolin was 392.3/603.9 and 262.0/401.1 mg/L, respectively, and similar to the episomal strain (334.3/695.9 mg/L). The intermediate product cinnamic acid is toxic to yeast and will significantly inhibit growth. Therefore, when integrated with Module IA genes, the strain Y621 only could accumulate 62.0 mg/L cinnamic acid and 34.5 mg/L *p*-coumaric acid from glucose (Fig. 7C).

The strains C901 and Y732 both simultaneously integrated three module genes at three different Ty sites under different selective markers. The accumulation of cinnamic acid, *p*-coumaric acid, naringenin, eriodictyol and taxifolin in C901 and Y732 were 0/11.9/13.4/22.7/50.9 and 122.4/39.7/1.4/60.7/173.6 mg/L from glucose, respectively (Fig. 7D). The *de novo* strains C901 and Y732 were analyzed through RT-qPCR to verify the copy numbers of pathway genes. The Module IA/II/III gene copy numbers in C901 and Y732 were 8/5/7 and 2/3/16, respectively (Fig. 7E). Higher copy numbers of Module IA did not lead to a higher concentration of taxifolin. This may be caused by an excessive accumulation of the toxic compound cinnamic acid in the early stage of growth, thus only a low copy number of strains survive and can be selected. The maximum DNA fragment length that could be integrated is 8.4 kb (Fig. 7E). However, if selective marker sequences were included, the total length could be up to 12 kb. This confirmed that the toolkit could achieve high-copy integrated expression of long-pathway genes in the genome of *S. cerevisiae*.

While the best strain Y732 was then fermented at 5-L bioreactor. The yield of taxifolin was 277.6 mg/L, exhibiting a mere 59.9 % increase from the level achieved in shake flasks (Fig. 7F). Unlike fermentation in the shake flask, only *p*-coumaric acid was significantly accumulated, with a concentration of 383.4 mg/L. All other intermediate products remained below 10 mg/L throughout the fermentation process.

## 4 Discussion

*S. cerevisiae* is an excellent chassis for the functional expression of eukaryotic genes and pathways. Long metabolic pathways of up to 30 genes could be reconstructed heterologously to obtain the production of target compounds, such as vitamin A [33], vinblastine [34] and protein [35]. However, it is hard to achieve the extra-high overexpression of genes and proteins, as in *Escherichia* *coli* or *Pichia* *pastoris*, due to a lack of efficient expression toolkits and manipulation methods [36,37]. Overexpression of genes is one of the key limiting factors for achieving industrial applications [38]. By using the multiple integration toolkit reported here, the copy number of heterogenous genes could easily reach 19. In our recent work, multiple integration of genes using the described strategy have already been applied in pathway reconstruction and have significantly improved 7-dehydrocholesterol [39], *ent*-copalo [40] and squalene [41] accumulation. The production of phiYFP was 1.6 g/L after only 24 h cultivation without any extra optimization by either metabolic engineering or culture conditions. This opens a new door to the efficient production of proteins in *S. cerevisiae*. Similar to the T7 system for *E. coli* and the AOX system for *P. pastoris* [42], the multiple integration toolkit and manipulation method described here could efficiently achieve the massive overexpression of genes, proteins, and even circularized mRNAs in *S. cerevisiae*.

Previous work has verified the integration ability of three auxotrophic selective markers *KlLEU2*, *KlURA3* and *SpHIS5* at five Ty sites. Only *KlURA3*deg showed perfect performance [9]. Here, nine selective markers (four auxotrophic and five resistance selective markers) were introduced, and the results verified that it is easy to obtain colonies with high integration copy numbers using the selective markers *ScTRP1*_AAG_^anti^deg, *KlLEU2*_GUG_deg, *KlURA3*deg, *SpHIS5*deg, *natMX*deg and *hphMX*deg at almost all five Ty sites. The integration ability of all selective markers at five Ty sites was also recorded and analyzed, the results showed that Ty2Cons, Ty3Cons and Ty4Cons sites could have a relatively higher integration copy number than Ty1Cons1 and Tyc1Cons2. This may be because the LTR sequences of Ty1Cons1 and Ty1Cons2 display a broad range of sequence diversity, whereas several Ty2, Ty3 and Ty4 LTR sequences share >95 % nucleotide identity within each element family [8,9,14].

This toolkit can be further improved and expanded. More specific selective markers could be developed for further integrative use, such as *THR4* [43], *ARG8* [44] and *dsdAMX* [45], if necessary. A variety of *S. cerevisiae* strains have similar Ty elements in their genomes, so the toolkit could also be applied to these strains, such as BY4741, FY4 and CEN.PK strains. The multiple integration of target genes could be a useful tool for achieving high-level stable expression of target genes in microorganisms. However, retrotransposons/transposons exist widely in many organisms. With bioinformatic analysis, retrotransposon/transposon elements may also be developed in other species, such as *Escherichia* [46], *Bacillus* [47], *Aspergillus* [48] and *Yarrowia* [49]. Unlike regulation strategies commonly used, such as enzyme rational design, pathway metabolic engineering and promoter engineering [50], the multiple integration strategy described here was simple and direct.

However, the gene copy number is not the only factor affecting protein and chemical accumulation. Toxic proteins such as lactoferrin and toxic chemicals such as the cinnamic acid will dramatically decrease yeast growth rate and only low copy number strains could be obtained after transformation. The balanced expression of genes is also important for chemicals and the efficiency of protein accumulation [51,52]. Therefore, only bottleneck genes are recommended for integrative overexpression instead of all pathway genes [25]. The genes resulting in toxic chemical accumulation can be integrated last, or after downstream genes have been integrated. The use of inducible promoters to control toxic genes and induce these genes after multiple integrations is also an alternative strategy to avoid toxic effects. The previous gene multiple integration leads to a decrease in cell growth and transformation efficiency, resulting in a situation where the earlier integration genes had high copy numbers, and the later genes had lower copy numbers. Therefore, more systems metabolic engineering strategies and fermentation process optimizations should be applied after genes multiple integration for the efficient production of chemicals and proteins [53]. When using large DNA fragments for integration, the integration efficiency will be significantly reduced. It can be improved by increasing the DNA fragment concentration and purity, and prolonging the plate culture time. Alternatively, large DNA fragments can be divided and individually integrated into different Ty sites to achieve high-copy integration.

*S. cerevisiae* is the most investigated eukaryotic microorganism for both microbial physiology and metabolic engineering research. The clearly demonstrated metabolic pathways, gene regulation networks, and a large amount of data on function-gene relationships make *S. cerevisiae* the most commonly used host for achieving the production of almost all of the useful proteins, nucleotides, fatty acids, and small molecular compounds, either native or heterologously introduced [54,55]. The expression level of both domestic and heterologous genes is vital in achieving the high accumulation of these useful compounds [56]. Combined with the Ty element-based multiple integration toolkit, the expression level of key genes could be further improved. Besides the mining of currently available expression elements, such as promoters, integration sites and terminators, designing and synthesizing more efficient expression elements could not only help in the understanding of mechanisms between the function and nucleotide sequences but also achieve more efficient expression levels than that obtained from nature.

## CRediT authorship contribution statement

**Song Gao:** Writing – original draft, Funding acquisition, Formal analysis, Data curation. **Weizhu Zeng:** Data curation. **Dong Li:** Data curation. **Sha Xu:** Writing – review & editing, Supervision, Funding acquisition, Formal analysis. **Jingwen Zhou:** Writing – review & editing, Writing – original draft, Project administration, Funding acquisition, Formal analysis, Data curation.

## Conflicts of interests

The authors declare that they have no competing interests.
