# Supplementary material for: Ty retrotransposon element based multiple integration toolkit for Saccharomyces cerevisiae
Source: Synth Syst Biotechnol. 2025 Apr 23;10(3):887–96. doi: 10.1016/j.synbio.2025.04.011 (PMC12083897; doi:10.1016/j.synbio.2025.04.011)
Supplement: Multimedia component 1 [file mmc1.docx]

# SUPPLEMENTARY INFORMATION

# Ty retrotransposon element based multiple integration toolkit for *Saccharomyces cerevisiae*

Song Gao^1,2,3,4^, Weizhu Zeng^1,3,4^, Dong Li^1,3,4^, Sha Xu^4^, Jingwen Zhou^1,3,4*^

^1^ Engineering Research Center of Ministry of Education on Food Synthetic Biotechnology, Jiangnan University, 1800 Lihu Road, Wuxi, Jiangsu 214122, China;

^2^ School of Food Science and Technology, Jiangnan University, 1800 Lihu Road, Wuxi, Jiangsu 214122, China;

^3^ Science Center for Future Foods, Jiangnan University, 1800 Lihu Road, Wuxi, Jiangsu 214122, China;

^4^ Key Laboratory of Industrial Biotechnology, Ministry of Education and School of Biotechnology, Jiangnan University, 1800 Lihu Road, Wuxi, Jiangsu 214122, China;

* Correspondence to:

Jingwen Zhou

Science Center for Future Foods, Jiangnan University, 1800 Lihu Rd, Wuxi, Jiangsu 214122, China.

Phone: +86-510-85918310, Fax: +86-510-85918310

E-mail: zhoujw1982@jiangnan.edu.cn

# SUPPLEMENTARY METHODS

## Plasmid and strain construction

The plasmids were constructed as follows:

Two DNA fragments were amplified with primer pairs Tvector-F/Tvector-R and M13-F/M13-R, respectively, with pMD19T-Simple as the template. The two DNA fragments were then purified and Gibson assembled, resulting in pcT-Simple. The size of pcT-Simple is 1818 bp, which is smaller than pMD19T-Simple.

The ORFs and promoters of *KlLUE2deg* and *KlURA3deg* were amplified from pcfB2990 and pcfB2989 with primer pairs LUE2-F/LEU2-R and URA3-F/URA3-R, respectively. The terminator T_TDH3_ was amplified from the S288c genome with primer pairs 2TTDH3-F/2TTDH3-R and 3TTDH3-F/3TTDH3-R, respectively. The vector sketch from pcT-simple was amplified with primer pairs pT2-F/pT2-R and pT3-F/PT3-R. The PCR products were Gibson assembled to yield pT2 and pT3.

Promoters P_ADE6_, P_ZWF1_, P_ARO7_, P_PYC1_, P_ADE3_, P_YEF3_ and P_ERG1_ were amplified from pMD19T-XXX[1] with primer pairs ADE6-F/ADE6-R, ZWF1-F/ZWF1-R, ARO7-F/ARO7-R, PYC1-F/PYC1-R, ADE3-F/ADE3-R, YEF3-F/YEF3-R and ERG1-F/ERG1-R, respectively. The ORFs of *ScTRP1*^anti^, *SpHIS5*, *natMX*, *hphMX*, *KanMX*, *patMX* and *bleMX* were amplified from pU57-TRP1-anti, pUG27, pAG25, pAG32, pUG6, pAG31 and pUG66 with primer pairs TRP1-F/TRP1-R, HIS5-F/HIS5-R, nat-F/nat-R, hph-F/hph-R, Kan-F/Kan-R, pat-F/pat-R and ble-F/ble-R. Vector backbones were amplified from pT2 with primer pairs pT1-F/pT1-R, pT5-F/pT5-R, pT6-F/pT6-R, pT7-F/pT7-R, pT8-F/pT8-R, pT9-F/pT9-R and pT0-F/pT0-R. The PCR products were Gibson assembled to yield pT1, pT5, pT6, pT7, pT8, pT9 and pT0.

The Ty LTRs sequences, Ty1Cons1up/Ty1Cons1dn, Ty1Cons2up/Ty1Cons2dn, Ty2up/Ty2dn, Ty3up/Ty4dn and Ty4up/Ty4dn were amplified from pcfB2989, pcfB2988, pcfB2797, pcfB2990 and pcfB2796 with primer pairs Ty11up-F/Ty11up-R/Ty11dn-F/Ty11dn-R, Ty12up-F/Ty12up-R/Ty12dn-F/Ty12dn-R, Ty2up-F/Ty2up-R/Ty2dn-F/Ty2dn-R, Ty3up-F/Ty3up-R/Ty3dn-F/Ty3dn-R and Ty4up-F/Ty4up-R/Ty4dn-F/Ty4dn-R. The terminators T_RFC5_-T_POL30_/T_SEC13_-T_PNP1_, T_MTD1_-T_RPF2_/T_LEU2_-T_NFS1_, T_DSF1_-T_HXT13_/T_TIM21_-T_GSC2_, T_RRP12_-T_TAF3_/T_RNA14_-T_BUB2_ and T_ADH1_/T_CYC1_ were amplified from S288c genome with primer pairs TT11up-F/TT11up-R/TT11dn-F/TT11dn-R, TT12up-F/TT12up-R/TT12dn-F/TT12dn-R, TT2up-F/TT2up-R/TT2dn-F/TT2dn-R, TT3up-F/TT3up-R/TT3dn-F/TT3dn-R and TT4up-F/TT4up-R/TT4dn-F/TT4dn-R, respectively. The reporter gene were amplified from pY26-PGAL7-EGFP[1] with primer pairs 11E-F/11E-R, 12E-F/12E-R, 2E-F/2E-R, 3E-F/3E-R and 4E-F/4E-R. Vector backbones were amplified from pcT-simple with primer pairs pcT11E-F/pcT11E-R, pcT12E-F/pcT12E-R, pcT2E-F/pcT2E-R, pcT3E-F/pcT3E-R and pcT4E-F/pcT4E-R. The PCR products were Gibson assembled to yield pcT11EGFP, pcT12EGFP, pcT2EGFP, pcT3EGFP and pcT4EGFP.

The select marker *TRP1* expression boxes were amplified from pT1 with primer pairs mpT111-F/mpT111-R, mpT121-F/mpT121-R, mpT21-F/mpT21-R, mpT31-F/mpT31-R and mpT41-F/mpT41-R. The vector backbones were amplified from pcT11EGFP, pcT12EGFP, pcT2EGFP, pcT3EGFP and pcT4EGFP with primer pairs pcT111-F/pcT111-R, pcT121-F/pcT121-R, pcT21-F/pcT21-R, pcT31-F/pcT31-R and pcT41-F/pcT41-R. The PCR products were Gibson assembled to yield pcT111, pcT121, pcT21, pcT31 and pcT41. The other plasmids pcT112/pcT122/pcT22/pcT32/pcT42, pcT113/pcT123/pcT23/pcT33/pcT43, pcT115/pcT125/pcT25/pcT35/pcT45, pcT116/pcT126/pcT26/pcT36/pcT46, pcT117/pcT127/pcT27/pcT37/pcT47, pcT118/pcT128/pcT28/pcT38/pcT48, pcT119/pcT129/pcT29/pcT39/pcT49, and pcT110/pcT120/pcT20/pcT30/pcT40 were all Gibson assembled in the same way as mentioned above.

PCR products of pcT111-pcT41 amplified with primer pairs pT1A-F/pT1A-R were Gibson assembled and transformed into *E. coli* JM109 to yield pcT111A-pcT41A. The initiation codon of gene *TRP1* in pcT111A-pcT41A was replaced with AAG. PCR product of pcT31 amplified with primer pairs pT1G-F/pT1G-R was Gibson assembled and transformed into *E. coli* JM109 to yield pcT31G. In pcT31G, the initiation codon of gene *TRP1* was replaced with GUG. The PCR product of pcT112-pcT42 amplified with primer pairs pT2G-F/pT2G-R was Gibson assembled and transformed into *E. coli* JM109 to yield pcT112G-pcT42G. In pcT112G-pcT42G, the initiation codon of the *LEU2* gene was replaced with GUG. The PCR product of pcT42 amplified with primer pairs pT2A-F/pT2A-R was Gibson assembled and transformed into *E. coli* JM109 to yield pcT32A. In pcT32A, the initiation codon of the *LEU2* gene was replaced with AAG.

The promoter P_INO1_, gene *EcoaroL* and vector backbone were amplified from pMDT-PINO1[1], pMD-T122[2] and T-vector with primer pairs PINO1-F/PINO-R and aroL-F/aroL-R, pT100-F/pT100-R, the products were Gibson assembled to yield pT100. The vector backbone, gene expression cassettes P_GAL1.10_-*ARO4*^fbr^-*ARO7*^fbr^, P_GAL7_-*Fj*TAL and P_INO1_-*EcoaroL* were amplified from pCfB2803, pMD-T132[2], pMD-T101[2] and pT100 with primer pairs LL-F/LL-R, LL47f-F/LL47f-R, LLTAL-F/LLTAL-R and LLaroL-F/LLaroL-R, the products were Gibson assembled to yield pCfB4-47LL.

The promoter P_FBA1_, gene *Sm*PAL and vector backbone were amplified from pMDT-PFBA1[1], pMD-SmPAL[3] and T-vector with primer pairs PFBA1-F/PFBA1-R, PAL-F/PAL-R and pT101-F/pT101-R, the products were Gibson assembled to yield pT101. The promoter P_TDH1_, gene *Sm*C4H and vector backbone were amplified from pY26-PTDH1-EGFP[1], pMD-SmC4H[3] and T-vector with primer pairs PTDH1-F/PTDH1-R, C4H-F/C4H-R and pT102-F/pT102-R, the products were Gibson assembled to yield pT102. The vector backbone, gene expression castle P_GAL1.10_-*ARO4*^fbr^-*ARO7*^fbr,^ P_GAL7_-*Fj*TAL, P_INO1_-*EcoaroL*, P_FBA1_-*Sm*PAL, P_TDH1_-*Sm*C4H and P_INO1_-*EcoaroL* were amplified from pcT21, pMD-T132[2], pMD-T101[2], pT101, pT102 and pT100 with primer pairs LHL-F/LHL-R, LHL47f-F/LHL-47f-R, LLPAL-F/LLPAL-R, LLC4H-F/LLC4H-R and LLaroL-F/LLaroL-R, the products were Gibson assembled to yield pcT21-LHL. The taxifolin biosynthesis pathway genes and vector backbone were amplified from pY26-P5m4[4] and pCfB2803 with primer pairs tax-F/tax-R and P5m4-F/P5m4-R and the products were Gibson assembled to yield pCfB4-P05m4.

The gene expression cassettes and vector backbone were amplified from pcT21-LHL and pcT23 with primer pairs LHL-2F/LHL-2R and pcT23-2F/pcT23-2R, Gibson assembled to yield pcT23-LHL. The gene expression castle and vector backbone from pY26-P03[5] and pcT32G were amplified with primer pairs P03-2F/P03-2R and pcT32G-2F/pcT32G-2R, Gibson assembled to yield pcT32G-P03. The gene expression castle and vector backbone from pCfB4-P05m4 and pcT41A were amplified with primer pairs P5m4-2F/P5m4-2R and pcT41A-2F/pcT41A-2R, Gibson assembled to yield pcT41A-P5m4.

The fluorescent protein expression frames and vector backbones were amplified from pY26-PGAL7-phiYFP, pY26-PGAL7-mKOk, pY26-PGAL7-mKate2 and pcT23, pcT37, pcT41A with primer pairs YFP-F/YFP-R, KOK-F/KOF-R, Kate-F/Kate-R and pcT23E-F/pcT23E-R, pcT37E-F/pcT37E-R, pcT41AE-F/pcT41AE-R, the PCR products were Gibson assembled to yield pcT23-phiYEF, pcT37-mKOk and pcT41A-mKate2.

The vector backbones and 26s rDNA up and down homologous arms from pMDT-simple and C800 genome were amplified with primer pairs T-F/T-R, rDNAup-F/rDNAup-R and rDNAdn-F/rDNAdn-R, the PCR products were Gibson assembled and yield prT-simple. The selective markers, terminators and P_GAL7_-EGFP expression box from pcT111, pcT122G, pcT23, pcT31A, pcT45, pcT36 and pcT37 were amplifeid with primer pairs rTRP1-F/rTRP1-R, rLEU2-F/rLEU2-R, rURA3-F/rURA3-R, rTRP1AAG-F/rTRP1AAG-R, rHIS5-F/rHIS5-R, rnat-F/rnat-R and rhph-F/rhph-R, the vector backbone prT-simple was amplified with primer pairs RTRP1-F/RTRP1-R, RLEU2-F/RLEU2-R, RURA3-F/RURA3-R, RTRP1AAG-F/RTRP1AAG-R, RHIS5-F/RHIS5-R, Rnat-F/Rnat-R and Rhph-F/Rhph-R, the PCR products were Gibson assembled and yield plasmids prT111, prT122, prT23, prT34, prT45, prT36 and prT37.

The integration boxes were amplified from pcT112G, pcT122G, pcT23-phiYFP, pcT37-mKOk and pcT41A-mKate2 with primer pairs Ty11-inte-F/Ty11-inte-R, Ty12-inte-F/Ty12-inte-R, Ty2-inte-F/Ty2-inte-R, Ty3-inte-F/Ty3-inte-R and Ty4-inte-F/Ty4-inte-R, after transformation, strain C811E, C812E, C802Y, C803O and C804K were yielded. The plasmids pY26-PGAL7-EGFP, pY26-PGAL7-phiYFP, pY26-PGAL7-mKOk and pY26-PGAL7-mKate2 were transformed into C800, resulting in strains C800E, C800Y, C800O and C800K. The integration boxes for strain C8011, C803, C805, C857 and C901 construction were from pCfB4-47LL, pcT21-LHL, pCfB4-P05m4 and pcT23-P03. The integration boxes for strain Y543, Y621 and Y732 construction were from pcT23-LHL, pcT32G-P03 and pCT41A-P5m4.

All integration used the following primer pairs for Ty sites Ty11-inte-F/Ty11-inte-R, Ty2-inte-F/Ty2-inte-R, Ty3-inte-F/Ty3-inte-R and Ty4-inte-F/Ty4-inte-R. All integration for 26s rDNA sites used the primer pair rDNA-F/rDNA-R.

## RT-qPCR primers

The gene *ACT1* was used as the reference gene. Primer pairs qaroL-F/qaroL-R, qCHS-F/qCHS-R, qF3ʹH-F/qF3ʹH-R, qFjTAL/qFjTAL, qphiYFP-F/qphiYFP-R, qmKOk-F/mKOk-R, qmKate2-F/qmKate2-R, qACT1-F/qACT1-R and qEGFP-F/qEGFP-R were used for RT-qPCR amplification.

# SUPPLEMENTARY FIGURE


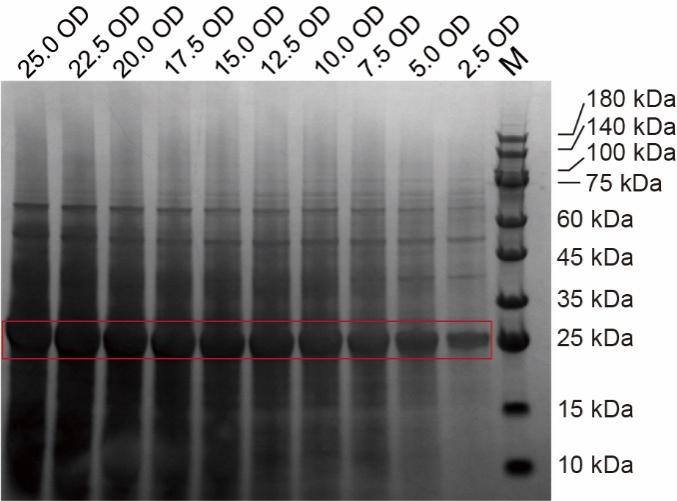


**Figure S1. The percentage of phiYFP to total protein production.**

The proteins were extracted from strain C802Y through high-pressure homogenization. A series of cell density (OD_600_) measurements were performed by SDS-PAGE, and the injection volume was 10 μL. The solid bands at around 25 kDa are the target protein. The protein yield of phiYFP accounted for 48.8% of the total protein yield based on a 2.5 OD injection well. M: marker. The target protein phiYFP is marked with red boxes.

# SUPPLEMENTARY TABLES

## Table S1 Plasmids used in this study

| **Plasmids** | | **Description** | **Sources** |
| --- | --- | --- | --- |
| pcfB2989 | | Ty1Cons1; *KlURA3*deg | [6] |
| pcfB2988 | | Ty1Cons2; *KlURA3*deg | [6] |
| pcfB2797 | | Ty2Cons; *KlURA3*deg | [6] |
| pcfB2990 | | Ty3Cons; *KlLEU2*deg | [6] |
| pcfB2796 | | Ty4Cons; *KlURA3*deg | [6] |
| pcfB2803 | | Ty4Cons; *KlLEU*deg | [6] |
| pUG27 | | *SpHIS5* | [7] |
| pUG6 | | *KanMX* | [7] |
| pUG66 | | *bleMX* | [7] |
| pAG25 | | *natMX* | [8] |
| pAG31 | | *patMX* | [8] |
| pAG32 | | *hphMX* | [8] |
| pcT-simple | | Amp | This study |
| prT-simple | | Amp; 26s rDNA | This study |
| pUC57-TRP1-anti | | *ScTRP1*^anti^ | This study |
| pCfB4-47LL | pCfB2803-Ty4Consup-P_GAL1,10_-*ARO4*^fbr^-*ARO7*^fbr-^P_GAL7_-*Fj*TAL-P_INO1_-*EcaroL*-T_SEC13_-T_PNP1_-P_LEU2_-*KlLEU2*deg-Ty4Consdn | This study |  |
| pcT21-LHL | pcT21-Ty2Consup-P_GAL1.10_-*ARO4*^fbr^-*ARO7*^fbr^-P_FBA1_-*Sm*PAL-P_TDH1_-*Sm*C4H-P_INO1_-*EcaroL*-P_GAL7_-EGFP-*ScTRP1*^anti^deg-Ty2Consdn | This study |  |
| pCfB4-P05m4 | pCfB2803-Ty4Consup-P_INO1_-*Sm*F3’H^D284N^-P_TDH1_-*Sm*CPR^I453V^-P_GAL7_-*Sm*F3H-*KlLEU2*deg | This study |  |
| pT820 | pMD19T-*gal80*up-P_GAL1.10_-*ScACC1*^S659A,S1157A^-*Se*ACS2^S641P^-*SpHIS5*-*gal80*dn | This study |  |
| pT100 | pMDT19-P_INO1_-*EcoaroL* | This study |  |
| pT101 | pMDT19-P_FBA1_-*Sm*PAL | This study |  |
| pT102 | pMDT19-P_TDH1_-*Sm*C4H | This study |  |
| pY26-TEF-GPD | | *KlURA3* | [1] |
| pY26-P03 | pY26-P_SED1_-*Pc*4CL-P_ERG20_-*Ph*CHS-P_PGK1_-*Ms*CHS | [1] |  |
| pY26-P5m4 | pY26-P_INO1_-*Sm*F3′H^D285N-^P_TDH1_-*Sm*CPR^I453V^-P_GAL7_-*Sm*F3H | [4-5] |  |
| pcT125-LL | pcT-Ty1Cons2up-T_RFC5_-T_POL30_-P_GAL1,10_-*ARO4*^fbr^-*ARO7*^fbr-^P_GAL7_-*Fj*TAL-P_INO1_-*EcaroL*-T_SEC13_-T_PNP1_-P_ZWF1_-*SpHIS5*deg-T_TDH3_-Ty1Cons1dn | This study |  |
| pcT23-LHL | pcT-Ty2consup-T_DSF1_-T_HXT13_-P_GAL1.10_-*ARO4*^fbr^-*ARO7*^fbr^-P_FBA1_-*Sm*PAL-P_TDH1_-*Sm*C4H-P_INO1_-*EcaroL*-T_TIM21_-T_GSC2_-P_URA_-*KlURA3*deg-T_TDH3_-Ty2Consdn | This study |  |
| pcT32-P03 | pcT-Ty3Consup-T_RRP12_-T_TAF3_-P_SED1_-*Pc*4CL-P_ERG20_-*Ph*CHS-P_PGK1_-*Ms*CHI-T_RNA14_-T_BUB2_-P_LEU2_-*KlLEU2*_GUG_deg-T_TDH3_-Ty3Consdn | This study |  |
| pcT44-P5m4 | pcT-Ty4Consup-T_ADH1_-P_INO1_-*Sm*F3’H^D284N^-P_TDH1_-*Sm*CPR^I453V^-P_GAL7_-*Sm*F3H-T_CYC1_-P_ADE6_-*ScTRP1*_AAG_^anti^deg-T_TDH3_-Ty4Consdn | This study |  |
| pcT23-phiYFP | | pcT-Ty2consup-T_DSF1_-T_HXT13_-P_GAL7_-phiYFP-T_TIM21_-T_GSC2_-P_URA_-*KlURA3*deg-T_TDH3_-Ty2Consdn | This study |
| pcT37-mKOk | | pcT-Ty3Consup-T_RRP12_-T_TAF3_-P_GAL7_-mKOk-T_RNA14_-T_BUB2_-P_PYC1_-*hphMX*deg-T_TDH3_-Ty3Consdn | This study |
| pcT44-mKate2 | | pcT-Ty4Consup-T_ADH1_-P_GAL7_-mKate-T_CYC1_-P_ADE6_-*ScTRP*_AAG_deg-T_TDH3_-Ty4Consdn | This study |
| pY26-EGFP | | pY26-P_GAL7_-EGFP | [1] |
| pY26-phiYFP | | pY26-P_GAL7_-phiYFP | This study |
| pY26-mKOk | | pY26-P_GAL7_-mKOk | This study |
| pY26-mKate2 | | pY26-P_GAL7_-mKate2 | This study |
| pT1 | | pMD19T-P_ADE6_-*ScTRP1*^anti^deg-T_TDH3_ | This study |
| pT2 | | pMD19T-P_LEU2_-*KlLEU2*deg-T_TDH3_ | This study |
| pT3 | | pMD19T-P_URA3_-*KlURA3*deg-T_TDH3_ | This study |
| pT5 | | pMD19T-P_ZWF1_-*SpHIS5*deg-T_TDH3_ | This study |
| pT6 | | pMD19T-P_ARO7_-*natMX*deg-T_TDH3_ | This study |
| pT7 | | pMD19T-P_PYC1_-*hphMX*deg-T_TDH3_ | This study |
| pT8 | | pMD19T-P_ADE3_-*KanMX*deg-T_TDH3_ | This study |
| pT9 | | pMD19T-P_YEF3_-*patMX*deg-T_TDH3_ | This study |
| pT0 | | pMD19T-P_ERG1_-*bleMX*deg-T_TDH3_ | This study |
| pcT11EGFP | | pcT-Ty1Cons1up-T_RFC5_-T_POL30_-P_GAL7_-EGFP-T_SEC13_-T_PNP1_-Ty1Cons1dn | This study |
| pcT12EGFP | | pcT-Ty1Cons2up-T_MTD1_-T_RPF2_-P_GAL7_-EGFP-T_LEU2_-T_NFS1_-Ty1Cons2dn | This study |
| pcT2EGFP | | pcT-Ty2Consup-T_DSF1_-T_HXT13_-P_GAL7_-EGFP-T_TIM21_-T_GSC2_-Ty2Consdn | This study |
| pcT3EGFP | | pcT-Ty3Consup-T_RRP12_-T_TAF3_-P_GAL7_-EGFP-T_RNA14_-T_BUB2_-Ty3Consdn | This study |
| pcT4EGFP | | pcT-Ty4Consup-T_ADH1_-P_GAL7_-EGFP-T_CYC1_-Ty4Consdn | This study |
| pcT111 | | pcT-Ty1Cons1up-T_RFC5_-T_POL30_-P_GAL7_-EGFP-T_SEC13_-T_PNP1_-P_ADE6_-*ScTRP1*^anti^deg-Ty1Cons1dn | This study |
| pcT121 | | pcT-Ty1Cons2up-T_MTD1_-T_RPF2_-P_GAL7_-EGFP-T_LEU2_-T_NFS1_-P_ADE6_-*ScTRP1*^anti^deg-Ty1Cons2dn | This study |
| pcT21 | | pcT-Ty2Consup-T_DSF1_-T_HXT13_-P_GAL7_-EGFP-T_TIM21_-T_GSC2_-P_ADE6_-*ScTRP1*^anti^deg-Ty2Consdn | This study |
| pcT31 | | pcT-Ty3Consup-T_RRP12_-T_TAF3_-P_GAL7_-EGFP-T_RNA14_-T_BUB2_-P_ADE6_-*ScTRP1*^anti^deg-Ty3Consdn | This study |
| pcT41 | | pcT-Ty4Consup-T_ADH1_-P_GAL7_-EGFP-T_CYC1_-P_ADE6_-Sc*TRP1*^anti^deg-T_TDH3_-Ty4Consdn | This study |
| pcT112 | | pcT-Ty1Cons1up-T_RFC5_-T_POL30_-P_GAL7_-EGFP-T_SEC13_-T_PNP1_-P_LEU2_-*KlLEU2*deg-Ty1Cons1dn | This study |
| pcT122 | | pcT-Ty1Cons2up-T_MTD1_-T_RPF2_-P_GAL7_-EGFP-T_LEU2_-T_NFS1_-P_LEU2_-*KlLEU2*deg-Ty1Cons2dn | This study |
| pcT22 | | pcT-Ty2Consup-T_DSF1_-T_HXT13_-P_GAL7_-EGFP-T_TIM21_-T_GSC21_-P_LEU2_-*KlLEU2*deg-Ty2Consdn | This study |
| pcT32 | | pcT-Ty3Consup-T_RRP12_-T_TAF3_-P_GAL7_-EGFP-T_RNA14_-T_BUB2_-P_LEU2_-*KlLEU2*deg-Ty3Consdn | This study |
| pcT42 | | pcT-Ty4Consup-T_ADH1_-P_GAL7_-EGFP-T_CYC1_-P_LEU2_-*KlLEU2*deg-T_TDH3_-Ty4Consdn | This study |
| pcT113 | | pcT-Ty1Cons1up-T_RFC5_-T_POL30_-P_GAL7_-EGFP-T_SEC13_-T_PNP1_-P_URA_-*KlURA3*deg-Ty1Cons1dn | This study |
| pcT123 | | pcT-Ty1Cons2up-T_MTD1_-T_RPF2_-P_GAL7_-EGFP-T_LEU2_-T_NFS1_-P_URA_-*KlURA3*deg-Ty1Cons2dn | This study |
| pcT23 | | pcT-Ty2Consup-T_DSF1_-T_HXT13_-P_GAL7_-EGFP-T_TIM21_-T_GSC2_-P_URA_-*KlURA3*deg-Ty2Consdn | This study |
| pcT33 | | pcT-Ty3Consup-T_RRP12_-T_TAF3_-P_GAL7_-EGFP-T_RNA14_-T_BUB2_-P_URA_-*KlURA3*deg-Ty3Consdn | This study |
| pcT43 | | pcT-Ty4Consup-T_ADH1_-P_GAL7_-EGFP-T_CYC1_-P_URA_-*KlURA3*deg-T_TDH3_-Ty4Consdn | This study |
| pcT115 | | pcT-Ty1Cons1up-T_RFC5_-T_POL30_-P_GAL7_-EGFP-T_SEC13_-T_PNP1_-P_ZWF1_-*SpHIS5*deg-Ty1Cons1dn | This study |
| pcT125 | | pcT-Ty1Cons2up-T_MTD1_-T_RPF2_-P_GAL7_-EGFP-T_LEU2_-T_NFS1_-P_ZWF1_-*SpHIS5*deg-Ty1Cons2dn | This study |
| pcT25 | | pcT-Ty2Consup-T_DSF1_-T_HXT13_-P_GAL7_-EGFP-T_TIM21_-T_GSC2_-P_ZWF1_-*SpHIS5*deg-Ty2Consdn | This study |
| pcT35 | | pcT-Ty3Consup-T_RRP12_-T_TAF3_-P_GAL7_-EGFP-T_RNA14_-T_BUB2_-P_ZWF1_-*SpHIS5*deg-Ty3Consdn | This study |
| pcT45 | | pcT-Ty4Consup-T_ADH1_-P_GAL7_-EGFP-T_CYC1_-P_ZWF1_-*SpHIS5*deg-T_TDH3_-Ty4Consdn | This study |
| pcT116 | | pcT-Ty1Cons1up-T_RFC5_-T_POL30_-P_GAL7_-EGFP-T_SEC13_-T_PNP1_-P_ARO7_-*natMX*deg-Ty1Cons1dn | This study |
| pcT126 | | pcT-Ty1Cons2up-T_MTD1_-T_RPF2_-P_GAL7_-EGFP-T_LEU2_-T_NFS1_-P_ARO7_-*natMX*deg-Ty1Cons2dn | This study |
| pcT26 | | pcT-Ty2Consup-T_DSF1_-T_HXT13_-P_GAL7_-EGFP-T_TIM21_-T_GSC2_-P_ARO7_-*natMX*deg-Ty2Consdn | This study |
| pcT36 | | pcT-Ty3Consup-T_RRP12_-T_TAF3_-P_GAL7_-EGFP-T_RNA14_-T_BUB2_-P_ARO7_-*natMX*deg-Ty3Consdn | This study |
| pcT46 | | pcT-Ty4Consup-T_ADH1_-P_GAL7_-EGFP-T_CYC1_-P_ARO7_-*natMX*deg-T_TDH3_-Ty4Consdn | This study |
| pcT117 | | pcT-Ty1Cons1up-T_RFC5_-T_POL30_-P_GAL7_-EGFP-T_SEC13_-T_PNP1_-P_PYC1_-*hphMX*deg-Ty1Cons1dn | This study |
| pcT127 | | pcT-Ty1Cons2up-T_MTD1_-T_RPF2_-P_GAL7_-EGFP-T_LEU2_-T_NFS1_-P_PYC1_-*hphMX*deg-Ty1Cons2dn | This study |
| pcT27 | | pcT-Ty2Consup-T_DSF1_-T_HXT13_-P_GAL7_-EGFP-T_TIM21_-T_GSC2_-P_PYC1_-*hphMX*deg-Ty2Consdn | This study |
| pcT37 | | pcT-Ty3Consup-T_RRP12_-T_TAF3_-P_GAL7_-EGFP-T_RNA14_-T_BUB2_-P_PYC1_-*hphMX*deg-Ty3Consdn | This study |
| pcT47 | | pcT-Ty4Consup-T_ADH1_-P_GAL7_-EGFP-T_CYC1_-P_PYC1_*-hphMX*deg-T_TDH3_-Ty4Consdn | This study |
| pcT118 | | pcT-Ty1Cons1up-T_RFC5_-T_POL30_-P_GAL7_-EGFP-T_SEC13_-T_PNP1_-P_ADE3_-*KanMX*deg-Ty1Cons1dn | This study |
| pcT128 | | pcT-Ty1Cons2up-T_MTD1_-T_RPF2_-P_GAL7_-EGFP-T_LEU2_-T_NFS1_-P_ADE3_-*KanMX*deg-Ty1Cons2dn | This study |
| pcT28 | | pcT-Ty2Consup-T_DSF1_-T_HXT13_-P_GAL7_-EGFP-T_TIM21_-T_GSC2_-P_ADE3_-*KanMX*deg-Ty2Consdn | This study |
| pcT38 | | pcT-Ty3Consup-T_RRP12_-T_TAF3_-P_GAL7_-EGFP-T_RNA14_-T_BUB2_-P_ADE3_-*KanMX*deg-Ty3Consdn | This study |
| pcT48 | | pcT-Ty4Consup-T_ADH1_-P_GAL7_-EGFP-T_CYC1_-P_ADE3_-*KanMX*deg-T_TDH3_-Ty4Consdn | This study |
| pcT119 | | pcT-Ty1Cons1up-T_RFC5_-T_POL30_-P_GAL7_-EGFP-T_SEC13_-T_PNP1_-P_YEF3_-*patMX*deg-Ty1Cons1dn | This study |
| pcT129 | | pcT-Ty1Cons2up-T_MTD1_-T_RPF2_-P_GAL7_-EGFP-T_LEU2_-T_NFS1_-P_YEF3_-*patMX*deg-Ty1Cons2dn | This study |
| pcT29 | | pcT-Ty2Consup-T_DSF1_-T_HXT13_-P_GAL7_-EGFP-T_TIM21_-T_GSC2_-P_YEF3_-*patMX*deg-Ty2Consdn | This study |
| pcT39 | | pcT-Ty3Consup-T_RRP12_-T_TAF3_-P_GAL7_-EGFP-T_RNA14_-T_BUB2_-P_YEF3_-*patMX*deg-Ty3Consdn | This study |
| pcT49 | | pcT-Ty4Consup-T_ADH1_-P_GAL7_-EGFP-T_CYC1_-P_YEF3_-*patMX*deg-T_TDH3_-Ty4Consdn | This study |
| pcT110 | | pcT-Ty1Cons1up-T_RFC5_-T_POL30_-P_GAL7_-EGFP-T_SEC13_-T_PNP1_-P_ERG1_-*bleMX*deg-Ty1Cons1dn | This study |
| pcT120 | | pcT-Ty1Cons2up-T_MTD1_-T_RPF2_-P_GAL7_-EGFP-T_LEU2_-T_NFS1_-P_ERG1_-*bleMX*deg-Ty1Cons2dn | This study |
| pcT20 | | pcT-Ty2Consup-T_DSF1_-T_HXT13_-P_GAL7_-EGFP-T_TIM21_-T_GSC2_-P_ERG1_-*bleMX*deg-Ty2Consdn | This study |
| pcT30 | | pcT-Ty3Consup-T_RRP12_-T_TAF3_-P_GAL7_-EGFP-T_RNA14_-T_BUB2_-P_ERG1_-*bleMX*deg-Ty3Consdn | This study |
| pcT40 | | pcT-Ty4Consup-T_ADH1_-P_GAL7_-EGFP-T_CYC1_-P_ERG1_-*bleMX*deg-T_TDH3_-Ty4Consdn | This study |
| pcT111A | | pcT-Ty1Cons1up-T_RFC5_-T_POL30_-P_GAL7_-EGFP-T_SEC13_-T_PNP1_-P_ADE6_-*ScTRP1*_AAG_^anti^deg-Ty1Cons1dn | This study |
| pcT112A | | pcT-Ty1Cons2up-T_MTD1_T_RPF2_-P_GAL7_-EGFP-T_LEU2_-T_NFS1_-P_ADE6_-*ScTRP1*_AAG_^anti^deg-Ty1Cons2dn | This study |
| pcT21A | | pcT-Ty2Consup-T_DSF1_-T_HXT13_-P_GAL7_-EGFP-T_TIM21_-T_GSC2_-P_ADE6_-*ScTRP1*_AAG_^anti^deg-Ty2Consdn | This study |
| pcT31A | | pcT-Ty3Consup-T_RRP12_-T_TAF3_-P_GAL7_-EGFP-T_RNA14_-T_BUB2_-P_ADE6_-*ScTRP1*_AAG_^anti^deg-Ty3Consdn | This study |
| pcT41A | | pcT-Ty4Consup-T_ADH1_-P_GAL7_-EGFP-T_CYC1_-P_ADE6_-*ScTRP1*_AAG_^anti^deg-T_TDH3_-Ty4Consdn | This study |
| pcT34G | | pcT-Ty3Consup-T_RRP12_-T_TAF3_-P_GAL7_-EGFP-T_RNA14_-T_BUB2_-P_ADE6_-*ScTRP1*_GUG_^anti^deg-Ty3Consdn | This study |
| pcT112G | | pcT-Ty1Cons1up-T_RFC5_-T_POL30_-P_GAL7_-EGFP-T_SEC13_-T_PNP1_-P_LEU2_-*KlLEU2*_GUG_deg-Ty1Cons1dn | This study |
| pcT122G | | pcT-Ty1Cons2up-T_MTD1_-T_RPF2_-P_GAL7_-EGFP-T_LEU2_-T_NFS1_-P_LEU2_-*KlLEU2*_GUG_deg-Ty1Cons2dn | This study |
| pcT22G | | pcT-Ty2Consup-T_DSF1_-T_HXT13_-P_GAL7_-EGFP-T_TIM21_-T_GSC21_-P_LEU2_-*KlLEU2*_GUG_deg-Ty2Consdn | This study |
| pcT32G | | pcT-Ty3Consup-T_RRP12_-T_TAF3_-P_GAL7_-EGFP-T_RNA14_-T_BUB2_-P_LEU2_-*KlLEU2*_GUG_deg-Ty3Consdn | This study |
| pcT42G | | pcT-Ty4Consup-T_ADH1_-P_GAL7_-EGFP-T_CYC1_-P_LEU2_-*KlLEU2*_GUG_deg-T_TDH3_-Ty4Consdn | This study |
| pcT32A | | pcT-Ty3Consup-T_RRP12_-T_TAF3_-P_GAL7_-EGFP-T_RNA14_-T_BUB2_-P_LEU2_-*KlLEU2*_AAG_deg-Ty3Consdn | This study |
| prT111 | | prT-26srDNAup-T_RFC5_-T_POL30_-P_GAL7_-EGFP-T_SEC13_-T_PNP1_-P_ADE6_-*ScTRP1*^anti^deg-26srDNAdn | Derived from pcT111 |
| prT112 | | prT-26srDNAup-T_MTD1_-T_RPF2_-P_GAL7_-EGFP-T_LEU2_-T_NFS1_-P_LEU2_-*KlLEU2*_GUG_deg-26srDNAdn | Derived from pcT122G |
| prT23 | | prT-26srDNAup-T_DSF1_-T_HXT13_-P_GAL7_-EGFP-T_TIM21_-T_GSC2_-P_URA_-*KlURA3*deg-26srDNAdn | Derived from pcT23 |
| prT34 | | prT-26srDNAup-T_RRP12_-T_TAF3_-P_GAL7_-EGFP-T_RNA14_-T_BUB2_-P_ADE6_-*ScTRP1*_AAG_^anti^deg-26srDNAdn | Derived from pcT31A |
| prT45 | | prT-26srDNAup-T_ADH1_-P_GAL7_-EGFP-T_CYC1_-P_ZWF1_-*SpHIS5*deg-T_TDH3_-26srDNAdn | Derived from pcT45 |
| prT36 | prT-26srDNAup-T_RRP12_-T_TAF3_-P_GAL7_-EGFP-T_RNA14_-T_BUB2_-P_ARO7_-*natMX*deg-26srDNAdn | Derived from pcT36 |  |
| prT37 | | prT-26srDNAup-T_RRP12_-T_TAF3_-P_GAL7_-EGFP-T_RNA14_-T_BUB2_-P_PYC1_-*hphMX*deg-26srDNAdn | Derived from pcT37 |

## Table S2 Strains used in this study

| **Strains** | **Description** | **Sources** |
| --- | --- | --- |
| CEN.PK2-1D | *MATα*; *ura3-52*; *leu2-3,112*; *trp1-289*; *his3Δ1*; *MAL2-8^C^*; *SUC2* | [9] |
| C800 | CEN.PK2-1D; Δ*gal80*::*KanMX* | [1] |
| C850 | CEN.PK2-1D; Δ*gal80*::*SpHIS5* | This study |
| C887 | CEN.PK2-1D; Δ*gal80*::*KanMX*-P_GAL7_-EGFP | This study |
| C100 | C800; Δ*tpo1*::*Sm*F3´H-P_INO1_-P_TDH1_-*Sm*CPR |  |
| Y200 | CENPK2-1D, Δ*pdc5*::P_GAL7_-*Fj*TAL, *trp1*::P_GAL1,10_-*Ph*CHS-*Ms*CHI-*ScURA3*, Δg*al80*::P_GAL1,10_-*Pc*4CL-*Eco*aroL-*SpHIS5*; Δ*aro10*::P_GAL1,10_-*ARO4*^fbr^-*ARO7*^fbr^-*KanMX* | [1] |
| C811E | C800; Ty1Cons1::P_GAL7_-EGFP-*KlLEU2*_GUG_deg | pcT112G |
| C812E | C800; Ty1Cons2::P_GAL7_-EGFP-*KlLEU2*_GUG_deg | pcT122G |
| C802Y | C800; Ty2Cons::P_GAL7_-phiYFP-*KlURA3*deg | pcT23-phiYFP |
| C803O | C800; Ty3Cons::P_GAL7_-mKOk-*hphMX*deg | pcT37-mKOk |
| C804K | C800; Ty4Cons::P_GAL7_-mKate2-*ScTRP1*_AAG_^anti^deg | pcT41A-mKate2 |
| C800E | C800; pY26-EGFP | pY26-EGFP |
| C800Y | C800; pY26-phiYFP | pY26-phiYFP |
| C800O | C800; pY26-mKOk | pY26-mKOk |
| C800K | C800; pY26-mKate2 | pY26-mKate2 |
| C800P | C800; pY26-TEF-GPD | pY26-TEF-GPD |
| C8011 | C800; Ty4::P_GAL1,10_-*ARO4*^fbr^-*ARO7*^fbr-^P_GAL7_-*Fj*TAL-P_INO1_-*EcaroL*-T_SEC13_-T_PNP1_-P_LEU2_-*KlLEU2*deg | pCfB4-47LL |
| C803 | C800; Ty3Cons::P_SED1_-*Pc*4CL-P_ERG20_-*Ph*CHS-P_PGK1_-*Ms*CHI-*KlURA3*deg | [1] |
| C857 | C803; Δ*gal80*::P_GAL1.10_-*ScACC1*^S659A,S1157A^-*Se*ACS2^S641P^-*SpHIS5*; Ty4Cons:: P_INO1_-*Sm*F3´H^D284N^-P_TDH1_-*Sm*CPR^I453V^-P_GAL7_-*Sm*F3H-*KlLEU2*deg | pT820; pCfB4-P05m4 |
| C901 | C857; Ty2Coms::P_GAL1.10_-*ARO4*^fbr^-*ARO7*^fbr^-P_FBA1_-*Sm*PAL-P_TDH1_-*Sm*C4H-P_INO1_-*EcaroL*-P_GAL7_-EGFP-*ScTRP1*^anti^deg | pcT21-LHL |
| C805 | C800; Ty4Cons::P_INO1_-*Sm*F3’H^D284N^-P_TDH1_-*Sm*CPR^I453V^-P_GAL7_-*Sm*F3H-*KlLEU2*deg | pCfB4-P05m4 |
| Y543 | C800; Ty4Cons::P_INO1_-*Sm*F3’H^D284N^-P_TDH1_-*Sm*CPR^I453V^-P_GAL7_-*Sm*F3H-*ScTRP1*^anti^_AAG_deg | pcT41A-P5m4 |
| Y621 | Y543; Ty2cons::P_GAL1.10_-*ARO4*^fbr^-*ARO7*^fbr^-P_FBA1_-*Sm*PAL-P_TDH1_-*Sm*C4H-P_INO1_-*Ecaro*-*KlURA3*deg | pcT23-LHL |
| Y732 | Y621; Ty3Cons::P_SED1_-*Pc*4CL-P_ERG20_-*Ph*CHS-P_PGK1_-*Ms*CHI-*KlLEU2*_GUG_deg | pcT32G-P03 |

## Table S3 Sequences of key genes, promoters, terminators and LTRs

| **Genes** | **Sequences (5′-3′)** |
| --- | --- |
| *Sm*PAL | atgggcagcagccatcatcatcatcatcacagcagcggcctggtgccgcgcggcagccatatgatggatcaatataccaatggacattccactagtaagatgaatcatgatgatcatagtagtttatgccagactactacgacggacccattgaattggggtgtggctgcggcggcgttaagcggcagccacctcaacgaggtgaagaagatggtggaggagtatcggaatccggtggtgaggttgggtggcgaaacgctgactattggtcaggtagcagcggtcgccaccactagggacgttcaggttgagttatcggaatcttctcgtgccggagttacagccagcagcgattgggtgatggatagcatgaaaagaggaggagacacctatggtgtcaccaccggtttcggtgccacctctcaccggagaacaaaggaaggtggtgctcttcaacaagagctcattagattcttgaacgccggaatcttcggtaccggaacggaatccgaccatacgctgccgcaatccaccacaagagccgccatgttggtcagaatcaacaccctcctccaaggctactccggcatccgattcgaaatcttagaagccatcactaaatttctcaaccacaacatcaccccatgcctacccttacgtggcactatcacagcctccggtgacctggtcccactctcctacattgccggaatcttgaccggtcgtcacaattccaaggccgtcggccccaccggagaattactcgatgccgccaaatctttcgatcgtgccggtatcgatactggatttttcgagctacaaccgaaagaaggacttgcgttagtgaacgggaccgccgtggggtccggtatggcttcggtggttctttttgaagctaatatcctagcggttttatcggaggttttatcggccatttttgcggaagttatgcaaggaaaacctgagtttacggatcatctgacccataaactcaagcatcaccccggccagattgaggccgcggcgatcatggagcacattcttgatggtagctcctacgtaaaagaagcacaaaagatgcacgaaatggatccgcttcaaaaaccaaagcaagatcgttacgctcttcgtacgtcaccgcaatggctcggtccgcttatcgaggtgatacgtacatcgacgaaatcgatcgagagagagattaattcggttaatgataaccctttaattgatgtttctcgaaataaggcacttcatggagggaattttcagggtaccccaataggagtctcgatggataatacccgattagccgttgcatccatcgggaagcttttgtttgcgcagttttccgagcttgtgaatgatttttataacaatgggcttccatccaatctttctggaagccgtaatccaagtttggattacggcttcaaaggagcagagattgcgatggcatcctactgctcggaacttcagttcctcgcaaaccctgtaacgagtcacgttcaaagcgcagagcaacataaccaggatgtgaactcattagggttgatttcttcaaggaaaacggccgaagcagttgagattttaaaactcatgtcatccacttacttagtggcactatgccaagcagtcgacttgaggcacttagaagaaaacctcaagtcgactgtcaagaacgttgttagccaggtggccaagaaagtcttgaccaccggccacaatggccagcttcacccatctcgcttttgcgaaaaggatttgctcaaagtggtcgaccgtgagcacatcttttcgtacatcgatgacccgtgtagtgcaacttaccctctaatgcaaaaattacgacaagttatagtcgatcacgcgctcgcaaacggtgaggccgaaatggactcgagtacgtcaatcttccaaaagatcggggctttcgagcaagaactcgaaacccttttgccaaaagaagtcgaaagcactcgggtcgatcacgaaggtggaaagttagcgatttttaacagaatcgaagaatgtcgatcatacccgttgtataagtttgtaaggatggaactcggaaccggctatttgaccggcgagaagacggtttcaccgggagaggagtttgagaaggtgttctcggcgatatgtgccggaaaattgatggatccattgttggattgtttgaagatgtgggacggaaagccacttccaatttcataagcggccgcactcgagcaccaccaccaccaccactgagatccggctgctaacaaagcccgaaaggaagctgagttggctgctgccaccgctgagcaataactagcataaccccttggggcctctaaacgggtcttgaggggttttttgctgaaaggaggaactatatccggat |
| *Sm*C4H | atggatcttctccttttggagaaagctcttgtagggctcttcgtagccattttaggagcgatcttcatatctaagttacgtggaaagcgtttcaagctcccgccgggaccaattccggtaccgattttcggaaactggcttcaagtcggcgatgatctcaaccaccggaacttaacagatctagccaagaagttcggccagatcttccttctccgtatgggccaacggaacctcgtcgtcgtatcatcgccggatctcgccaaggaagtcctccacacacaaggcgtggagttcggatctcgaactagaaacgtcgtgttcgacattttcacagggaaaggacaagatatggtgtttacggtttacggcgagcactggcggaagatgcggaggatcatgacggttccgtttttcaccaataaagttgttcagcagtacaggttcggatgggaggcggaggcggcggcggtggtggaggatgtgaagaagaatccggcagcagcgacggaagggattgtgatcaggagacggttacagctgatgatgtataacaatatgttcagaattatgtttgatagaaggttcgagagtgaggacgatcctttgttcttgaagctcaaggcgttgaatggggagaggagtcgattggcacagagcttcgattacaactatggcgatttcatcccaattttgaggccgtttttgaaaggttatttgaagatgtgcaaagaagtcaaagagaagaggttgcagctattcaaggattacttcgttgatgaaaggaagaagatgggaagcataaaaaccatggacaacaaccaaatcaaatgtgcaattgatcatatacttgaagctcaggacaaaggagagatcaacgaggacaatgtcctttacatcgttgagaatatcaatgttgccgcaatcgaaaccaccctctggtccatcgaatggggaatcgcggaactcgtgaaccaccctgaaatccaatcgaaactgagacacgaactcgacaccaaactcggacccggagtccaagtcaccgaaccagacatccaaaagcttccatacctccaagccgtggttaaggagactctccgccttcggatggctatcccgctcctggtcccacacatgaacctccacgacgccaagcttaacggctacgacattccagccgaaagcaagatcttggtcaacgcctggtggctagccaacaaccccgaacaatggaagaaacccgatgaattccgacccgaaagattcttcgaagaagaaagccacgtggaggctaacggaaatgatttccgttacttgccgtttggagtcgggagaaggagttgtcccgggattatccttgcgttaccgatcttggggataacgatcgggcgattggtgcagaatttcgagctattgccgccaccggggatgtcgaagatcgatgtgaaggagaaaggtggacagtttagtttgcatattttgaatcattccaccgttgttgctaaaccaagatcattgtga |
| *Sm*F3′H | atgactatcctacccctgctactctacgcctccataactggtttactaatctatgtattgcttaacctacgcaccacccctcgttctaaccacctcccactcccacccggcccaaccccatggccaatcatcggaaacttacctcatcttggaagaataccgcaccatgcgctggcggccatggctacaaagtacggcccgttgatgcatctccggctcggcgtcgttgacgtggtggtggcggcgtctgcgtcggtggcggcacagtttttgaaggttcatgacgccaatttcgcgagtaggccgccgaactccggcgcgaaacacatcgcgtataattatcaggatctggtgtttgcaccttatggtcagaaatggcggatgcttaggaagatttgctccgtgcatctgttctctaacaaagcactcgatgatttccgtcacgttcgtcaggaggaggtggcgattctggtgcgcgctttggccggagccggtcgatctacggcggcggcgttaggtcaactacttaacgtttgcaccacaaacgcgttggcacgagtgatgttaggtcggagagtgttcgtggacggaagtgaaggcaatcgagacgcggatgaattcaaggatatggtggttgaagtgatggtattggccggagaattcaacatcggcgacttcattccggcgcttgattggctggatctgcaaagcgtgacgaagaagatgaagaaactccatctccgattcgattcgtttcttaacaaaatcctggaagaccatagaaatggaggtgacgtcacttcgggtaacgtggatttgctgagcacgttgatttcgctcaaggatgacgccgatggagagggcgggaagctttcagatatcgaaatcaaagctttgcttctgaatttattcactgcgggaacagacacatcatctagtacggtggaatgggcaatggctgaactcattcgccatccgcaattattgaagcaagcccaagaagaattggacactgttgttggtaaagaccggcttgtatccgaattggacctgagtagactaacattcctcgaagccattgtgaaggaaaccttcaggctccacccatcgaccccactctctttgccacggattgcatcagagagctgtgaagtcgatgggtattacattcctaagggaaccacacttcttgttaacgtgtgggccattgcccgagacccaaaaatgtggaccgacccgcttgaattccgacccacccggttcttgccgggaggtgaaaagccgaatgctaatgtaaagggaaatgattttgaaataataccgtttggggctggtcgaaggatttgtgcgggtatgagcctagggttacggatggttcagttgctcactgcgactctggttcatgcctttgattggaaattggctaacgggttagacccagagaagctcaatatggaagaagcttatgggttgacccttcaaagggctgcacccttgatggtgcacccaaccccacggttagctccccatttgtatgaaagcagtcaaggtttataa |
| *Sm*CPR | atgcaatcggactcgtctctggaaacgtcgtcgtttgatttgattaccgcagctcttaaggagaaagttattgatacagcaaacgcatctgatagtggagattcaacgatgcctccggctttggcgatgattttggaaaaccgtgagctgtttatgatgctgactacaacagtggctcttttgcttggatttattgtcgtttcgttctggaagagatcttctgagaagaagtcggctaaggatttggagctaccgaagatcgttgtgcctaagagacagcaggaacaggaggttgatgacggtaagaagaaggttacgattctttttggaacgcagaccggaacggcggaaggtttcgctaaggcactgttggaagaagctaaagcgcgatatgaaaaggcgacctttaaagtagtcgatttggatgattatgctgttgatgatgatgagtacgaagagaaactaaagaaggagtcatttgctttcttcttcttggctacatatggagatggtgagccaactgataatgctgccagattttataaatggtttacagagggaggtgagaaaggagtttggcttgaaaagcttcaatatggagtatttggccttggcaatagacaatacgagcatttcaacaagattgcaaaagaggttgacgatggtctcgcagagcagggtgcaaagcgccttgttccagttggccttggagatgatgatcaatccattgaagatgattttactgcatggaaagagttagtgtggcctgagttggatgaattgcttcgtgacgaggatgacaaaggcgttgctactccttacacagctgctattccggaataccgagttgtgtttcatgagaaacatgatacatctgctgaagatcaaattcagacaaatggtcatgctgttcatgatgctcaacatccatgcagatccaatgtggctgttaaaaaggagctccatacccctgaatctgatcgctcttgcacgcatctggaatttgacatctcacacactggactatcatacgaaactggggaccatgttggtgtctactgtgagaacttaagtgaagttgtggaggaggctgagaggttaataggtttaccatcggatacttatttctcagttcacacggataacgaagatggaacaccacttggtggagcttccttactacctcctttccctccatgcactttaagaaaagcattggctaattacgcagatgtattgacttctcccaaaaagtcggccttgattgctctagctgctcatgcttctgatcctactgaagctgaacgactaaaatttcttgcatctcctgctgggaaggatgaatattctcaatggattattgcaagccaaagaagcctgcttgaggtcatggaagctttcccatcggctaagcctccacttggggttttctttgcagctattgctccacgcttacagcctcgatactactctatttcttcctccccgaagatggcacctagcaggattcatgttacttgtgcattagtttatgagaaaacacctgcaggccgtctccataaaggaatctgttcaacctggatgaagaatgctgtgcctatgacggaaagtcaggattgcagctgggcacctattttcgttagaacgtctaacttcagacttcccactgatcctaaagttcctgttatcatgattggccctggaaccggattggctccgttcagaggttttcttcaagaaagattagctctgaaggaagccggaactgaactgggatcatccattttattcttcggatgtagaaatcgcaaagtggatttcatatatgagaatgaactgaaagactttgttgagaatggtgctgtttccgagcttattgttgccttctcccgtgaaggccccaataaggaatatgtgcaacataaaatgagcgatagggcttcggatctatggaacttgctttcggagggagcatatttatacgtttgtggtgatgccaaaggcatggctaaagatgtacaccggacccttcacacaattgtgcaagaacagggatctctagactcgtcaaaggcagagctgtatgtgaagaatctacaaatgtcaggaagatacctccgtgatgtttggtag |
| *Sm*F3H | atgcttgaaaacaggttcgttcgcgatgaagacgagcgtccaaaagtggcgtacaataattttagcaacgagattccggtgatctcacttgaaggtatcgacgatactagtagtagggcggagatttgcgagaagatcgttaaggcttgtgaagattggggggtttttcaggtggtggatcacgggatcgataatagattgttgacggagatgacgaggctcgccacggagttcttcatgatgccgccggaggagaaactccgatttgatatgagtggcgggaaaaaaggcggtttcattgtttccagccatcttcaaggagaaacggtgcaagattggagggagattgtaaccttcttctcgtacccaacaaaagcaagagactactctaggtggcccgataagcccaaagagtggagggcagttactgaggaatatagcaaggtgttaatgggcctggcctgcaagctactagaggtattgtctgaggcaatgggccttgagaaagaggccttgaccaaagcttgtgtagatatggaccaaaaggtggtggtcaattactatccaaaatgccctcatcccgacctcacgttgggcctgaaacgacatacggatccgggaacaatcacgttgttgcttcaggaccaagttggtgggcttcaggcgactcgtgatggtggtcaaagttggatcacagttcagccgattgaaggtgcttttgtggttaatcttggtgatcatggacattatttgagcaacgggaggttcaagaacgcagaccaccaagccgtggtgaactcaaacacgagccgactctccatagctacgtttcaaaaccctgcaccggatgcgattgtatacccgctgaaagtgaatgagggagataaatcgataatggaagaagctataactttcatggagatgtacaagaagaagatgggtcgagaccttgagttggctcggcttaagaagctagccaaggacaagcaacaagatttggagaaagagaagccaatcgagaatatatttgcttag |
| *ScTRP1*^anti^ | atgtcggtgataaactttacaggaagtagtggacccttggtgaaggtctgcggattacagagtacagaggcggcagagtgtgctctagatagtgatgccgacctactaggaataatatgcgttcccaacaggaagaggacaatagaccccgtgatagctaggaagataagttcactagtcaaagcctacaagaacagttcaggaacacccaagtacctagtaggagtttttaggaaccagcccaaggaagacgtactagcgctagtgaatgattatggaatagatatagttcaattacatggcgatgagtcatggcaagaatatcaggagttcttaggacttcccgtcataaagcggttggtatttcccaaggattgtaatatcctcctttccgccgcaagtcaaaagccccactcattcatacccctcttcgacagtgaggccggaggaacaggagagcttctagactggaacagtatatcggattgggtaggacgacaagagagtcccgagagtcttcatttcatgctagctggaggactaacacccgagaacgtcggagatgccctacgactaaatggagtaataggagtagacgttagtggaggggtagagacaaatggagtgaaggacagtaataaaatagctaatttcgtaaaaaatgcaaagaag |
| Degradation signal CL-1 (deg) | gcttgtaaaaattggttctcttctttgtctcatttcgttattcatttg |
| EGFP | atgggtaagggagaagaacttttcactggagttgtcccaattcttgttgaattagatggtgatgttaatgggcacaaattttctgtcagtggagagggtgaaggtgatgcaacatacggaaaacttacccttaaatttatttgcactactggaaagcttcctgttccttggccaacacttgtcactactcttacttatggtgttcaatgcttttcaagatacccagatcatatgaagcggcacgacttcttcaagagcgccatgcctgagggatacgtgcaggagaggaccatcttcttcaaggacgacgggaactacaagacacgtgctgaagtcaagtttgagggagacaccctcgtcaacagaatcgagcttaagggaatcgatttcaaggaggacggaaacatcctcggccacaagttggaatacaactacaactcccacaacgtatacatcatggcagacaaacaaaagaatggaatcaaagttaacttcaaaattagacacaacattgaagatggaagcgttcaactagcagaccattatcaacaaaatactccaattggcgatggccctgtccttttaccagacaaccattacctgtccacacaatctgccctttcgaaagatcccaacgaaaagagagaccacatggtccttcttgagtttgtaacagctgctgggattacacatggcatggatgaactatacaaataa |
| phiYFP | atgtctagtggagcactgttgttccacggaaagatcccatatgttgttgagatggagggaaatgttgatggacacacattctccattagaggtaaaggttatggagatgcaagtgttggtaaagttgatgcccaattcatctgcacaactggagatgtaccagttccatggtcaactttagtaacaacacttacttatggtgcacaatgcttcgccaaatatggtccagaattaaaggatttctacaagagttgcatgcctgaaggctatgtgcaggagcgtacaatcacatttgaaggggacggagtatttaaaactcgcgctgaagttacatttgaaaacggatctgtttataaccgagtcaaacttaatggacaaggatttaagaaagacggacatgtgcttggaaagaatcttgaattcaatttcacacctcattgtctttacatttggggagatcaggctaatcatggtttgaagtctgctttcaaaattatgcatgagattactggatcaaaagaagacttcattgttgcagaccacacccaaatgaacacacccattggtggtggaccagtccatgtccctgaataccatcatataacataccatgtcactctcagcaaagatgttactgatcacagggataacatgagcttggttgaaaccgtacgggctgtggattgcagaaaaacatatctttaa |
| mKOk | atgtctgttattaagccagaaatgaagatgagatattacatggatggttctgttaatggtcatgaattcactattgaaggtgaaggtactggtagaccatatgaaggtcatcaagaaatgactttgagagttactatggctgaaggtggtccaatgccattcgctttcgatttggtttctcatgttttctgttacggtcatagagtttttactaagtacccagaagaaattccagattacttcaagcaagcttttccagaaggtttgtcttgggaaagatctttggaattcgaagatggtggttctgcttcagtttctgctcatatttctttgagaggtaacactttttaccataagtctaagttcactggtgttaattttccagctgatggtccaattatgcaaaatcaatctgttgattgggaaccatctactgaaaagattaccgcttctgatggtgttttgaagggtgacgttactatgtacttgaagttggaaggtggtggtaaccataagtgtcaattcaagaccacttacaaggctgctaaggaaattttggaaatgccaggcgatcattacattggtcatagattggttagaaagactgaaggtaacattactgaacaagttgaagatgctgttgcccattcttaa |
| mKate2 | atggtgagcgagctgattaaggagaacatgcacatgaagctgtacatggagggcaccgtgaacaaccaccacttcaagtgcacatccgagggcgaaggcaagccctacgagggcacccagaccatgagaatcaaggcggtcgagggcggccctctccccttcgccttcgacatcctggctaccagcttcatgtacggcagcaaaaccttcatcaaccacacccagggcatccccgacttctttaagcagtccttccccgagggcttcacatgggagagagtcaccacatacgaagacgggggcgtgctgaccgctacccaggacaccagcctccaggacggctgcctcatctacaacgtcaagatcagaggggtgaacttcccatccaacggccctgtgatgcagaagaaaacactcggctgggaggcctccaccgagaccctgtaccccgctgacggcggcctggaaggcagagccgacatggccctgaagctcgtgggcgggggccacctgatctgcaacttgaagaccacatacagatccaagaaacccgctaagaacctcaagatgcccggcgtctactatgtggacagaagactggaaagaatcaaggaggccgacaaagagacctacgtcgagcagcacgaggtggctgtggccagatactgcgacctccctagcaaactggggcacagatga |
| P_GAL7_ | tttgccagcttactatccttcttgaaaatatgcactctatatcttttagttcttaattgcaacacatagatttgctgtataacgaattttatgctattttttaaatttggagttcagtgataaaagtgtcacagcgaatttcctcacatgtagggaccgaattgtttacaagttctctgtaccaccatggagacatcaaaaattgaaaatctatggaaagatatggacggtagcaacaagaatatagcacgagccgcggagttcatttcgttacttttgatatcactcacaactattgcgaagcgcttcagtgaaaaaatcataaggaaaagttgtaaatattattggtagtattcgtttggtaaagtagagggggtaatttttcccctttattttgttcatacattcttaaattgctttgcctctccttttggaaagctatacttcggagcactgttgagcgaaggctcattagatatattttctgtcattttccttaacccaaaaataagggaaagggtccaaaaagcgctcggacaactgttgaccgtgatccgaaggactggctatacagtgttcacaaaatagccaagctgaaaataatgtgtagctatgttcagttagtttggctagcaaagatataaaagcaggtcggaaatatttatgggcattattatgcagagcatcaacatgataaaaaaaaacagttgaatattccctcaaaa |
| P_ADE6_ | ctgaacgtatcgagactcggttgtgtcgttatgctagcaatgtcctcacaggctccattccttctttcgctctattggatatcatcacagctattctccctggtgcaaaatatcatattaaattggatttatccttaccaacgatggtgaagctgacgcatagataggatatgtaattctacatcagcttgtaaataaacaaaaatgactttcaatatccttcaaccgttcctgactctttcctgctgacccgtttttccaaatttctcgtcgaacttgaaattgaaaaaaaaaaaaaaaaattgaatgaggactcattaaacagatgatgccgtaataaatgcaatatatcttgctatttaactctttctttctttgaaaaccttgacatacgtatttaaataattggctgtccctgcctcgaagtatatttctcttctacttttatcttagcgatatccctaagagtttaatcctcccaggtccataacaaaagaagtcaagttca |
| P_LEU2_ | gagctcgctgtgaagatcccagcaaaggcttacaaagtgttatctcttttgagacttgttgagttgaacactggtgttttcatcaaacttaccaaggacgtgtacccattgttgaaacttgtatcaccatatattgttatcggacaaccttcacttgcatctatccgttctttaatccaaaagagatctagaataatgtggcaaaggccagaagataaagaaccaaaagagataatcttgaatgacaacaatatcgttgaagagaaattaggtgatgaaggtgtcatttgtatcgaggatatcatccatgagatttcgacgttgggcgaaaatttctcgaaatgtactttcttcctattaccattcaaattgaacagagaagtcagtggattcggtgccatctcccgtttgaataaactgaaaatgcgcgaacaaaacaaggagactcgtcaaatttcaaacgctgccacggctccagttatccaagtagatatcgactcaatgatttccaagttgaattgattaactataaaaggaaaatatctgtacaatagacatcgggctcccattggccctacccacatatgtagaaatacattactctattcactactgcatttagttatgtttaacatttgatatagcagactaccgccaggcacaatatattccccttccctcttgccattcgctgtacttgtggtggattccaattcagcgcagtcacgtgctagtaatcaccgcatttttttcttttcctttcaggctaaaaccggttccgggcctgatccctgcactcattttctaacggaaaaccttcagaagcataactacccattccagtttagagtcatgacaggttcaacatcagatgcttcatatacttttatatattgaattatataaatatatctatgtactctaagtaagtacatctgctttaacgcattcctacatttgcttcgatttatttttattgttgatacctatttgaagaagtaaaaagtatcccacactacacagattatacc |
| P_URA3_ | ttttatttaggttctatcgaggagaaaaagcgacaagaagagatagaccatggataaactgattatgttctaaacactcctcagaagctcatcgaactgtcatcctgcgtgaagattaaaatccaacttagaaatttcgagcttacggagacaatcatatgggagaagcaattggaagatagaaaaaaggtactcggtacataaatatatgtgattctgggtagaagatcggtctgcattggatggtggtaacgcatttttttacacacattacttgcctcgagcatcaaatggtggttattcgtggatctatatcacgtgatttgcttaagaattgtcgttcatggtgacacttttagctttgacatgattaagctcatctcaattgatgttatctaaagtcatttcaactatctaagatgtggttgtgattgggccattttgtgaaagccagtacgccagcgtcaatacactcccgtcaattagttgcacc |
| P_ZWF1_ | gccgtcgaaaaggatctcgtctctgttgggagcacctggtaagtaaggtgtagttttgcacccgtgtacataagcgtgaaatcaccacaaactgtgtgtatcaagtacatagtgacatttaaataatagcaagaacaacaataatagtagcgctactggaagcaccacgtaatagtggaaaagaactggaaaaaccgctataagatgcatactccggcggtcttacgcggagatacaagcttccaacggtgctaaaagcccggtttcggctcggccggaggaggaagagagacgaaaaaaaaaaaaatgactaaaaaaaaaatggaatattattaatgtgggatttttggctcaaggtgtggtggccccttttctaagggtggcgaattcttcaatgtacggaaaactcgccaaggctatcccatatataagcaaactgtgggttcatctatataccgacacataacacctaaagtggcttcctcctgcccctctctcccttttctccactcacccctccttctcccccttccccctctccaattggctgtatagacagaaagagtaaatccaatagaatagaaaaccacataaggcaag |
| P_ARO7_ | tggattacatttgattcagtcatacacgaattatggtcttgatactgacaaattttccagattgaggcggttcttatggtttagaacttggggactttacaagtcgaaagaggatttagatagagaagccaagatcaatgaagaaatgatacgcaaactgaaagcagctaaatgaaatcacctattgcgccgctcgcggaatacaattactaaattttatatatattctttaaaaatgcatctatacattcgtttttccacgtataccaaattcgaaaaaagttgttaaaccatcgttttcacgttttttaatttttttttggttctctttttttttttttttcaatatcaactttttttcaaacttcgtgttgcatttcctttatcgtaaattttcaatggatctctataatcttcgaagttcgaagaaaagaagaaaaaaagtattgaaaagttgaaacatcgattccgttttgctaacaaatagcactcagcatcctgcataaaattggtataagat |
| P_ALD6_ | cgtatccaagccgaaacggcgctcgcctcatccccacgggaataaggcagccgacaaaagaaaaacgaccgaaaaggaaccagaaagaaaaaagagggtgggcgcgccgcggacgtgtaaaaagatatgcatccagcttctatatcgctttaactttaccgttttgggcatcgggaacgtatgtaacattgatctcctcttgggaacggtgagtgcaacgaatgcgatatagcaccgaccatgtgggcaaattcgtaataaattcggggtgagggggattcaagacaagcaaccttgttagtcagctcaaacagcgatttaacggttgagtaacacatcaaaacaccgttcgaggtcaagcctggcgtgtttaacaagttcttgatatcatatataaatgtaataagaagtttggtaatattcaattcgaagtgttcagtcttttacttctcttgttttatagaagaaaaaacatcaagaaacatctttaacatacacaaacacatactatcagaataca |
| P_ADE3_ | acgtgagctaaagcacagattgttggaaaagcaagcggacggccgcggagcgctgaacgtgattttccattgtatgttatcgcagcagcgcggaccgtctgcagcgatgctgctgcttcggtcgcttgacacggcggaactttctcgctgtcgtctatgggtgttgcgtggggggttctcgcgctggcaatccgtatacggtgacgacgagagcgttacggcgggttacctacccgatctgtggcgttgacaggtttacacaatcgcacgtgatcatatatttgccatgactcctcccagtgacaattttgttcttttttcctctttatcgctttcgtactatggtcagtcattcattcattatatacgcgctctccataacccgtaactttttattatatatagactcgtttacaatacaacgatagcgataccattcaattgaagttgtgagaccaggtaacgagacgaacacaactttacaagtcaaataagaaatc |
| P_YEF3_ | aataagcgccactatcagggaatagcaactttcccttctgtttcaatctttttacctattcctttttaaaagatatatatacattaaactccttctacaagtatatattttatacatatctacagggcgtatatatacataacattttaagataagcaagtgaatgttgattcccgtttcttagtcaacacttctttctattttacccggtcgttaccctattaaaaaaacaacttacaatcattgttcgccccttccatacttactgccactcgcaaaagggcccaaccagggcaattacgtatcaaaaaatcatgacaggctgggtaataaatattcgtgaagaaagaagaaattaaaaaaagaaacgaagaagcaaaaaaaagaaaagactccgtttaatcactttcaaccgcggtttatccggccccacccatgcataaccctaaattattagatcacttagcacgtgaaaaagaaacgtttttaatgttttttttttttttttctttttctttttttgcgttggtgaaaattttttcgcttcctcgagtataattatctcatctcatctttcatataagataagaagttttataaaaaccttttgcatcaaaattttgtagaatatctctttttcttacgctctctttctttccttaattgttttctaaagaaccgtgtatttttctagttcgaatccatcgataacattaaaag |
| P_ERG1_ | gtgaatggtatgaacatggacatgagcgtggttcagggcactctacgggatcgtggcgaatgggaatcgttctgcaagctcttctaccaaaccatcggcgaatttgcgtcgctttaatgcgatactgccgtagcgggccttcgtatagctcggccgagctcgtacaaaaggcaagcagtgtatcggacagagctgatataacacaatacgctcgtagtcgatgcatgccgtggctgctctcggtcgggtataagtcttagacaatagtcttacctcgcatgtataataaatcttttgtatttaatctattatatgtttctatgcttttttttcctattgttgtttgcttttccttttccttatttctttctagcttctaattttctttcttttttttttttttttcattgaaaattatatatatatatatatatcagaacaattgtccagtattgaacaatacaggttatttcgaacaattgaaaaaaaaaaatcacagaaaaacatatcgagaaaagggtc |
| T_RFC5_-T_POL30_ | caagtgtttttgacgaaagattatcacttggaaacaaagcaatattccatttggaagggttcatagcaaaagttatgtgctgtctagattaatgtaagatatgtcataaatactgtataagtcacacaaaaagctgatatttaacgcatcttagtctttattttctttgttatttattttcatttaaaacaaactttactgttttttttttgtttattatttttagtatacaactatatagataatttacatttattcttcgtcattaaatttaggagccaagaaaaactgtaggaacccactcttcaaatcaaattggaataaagcaggagcttcgctggag |
| T_SEC13_-T_PNP1_ | agagtatcaagaatttaaaatgaaacatctcaaaagaaaaaagaatgcaaatgagtctatcgacgaatttgaagggaaataaacgcataatgtacagtaacgtataacaattaaagatttgtggaagttttcaaaaacttttcaacttttttctttgttttttttttgcaacttcttatattaatattgtcatagatatttcttatacaaaaacaagcgaacaaaaataatcgacgtatatacaatagatatataagactgtttttcttcaatagaacaggcgaaattattctaccggccgaaggtacatcttcccgctatgtaataaatagaggtatttaagttataacaa |
| T_MTD1_-T_RPF2_ | agcttctctttacgttccaatgactggtaaagttaccattgcaatgttgttgagaaacatgttacgtttagtaaggaacgtagaactgtctaaagaaaaatagaacatttgtggctgttcaaataagcataggtaaaacagaatacataaaaataagggaaaaaaagaaagatcatttgtaatgtacttactacatacatatagaattctcctctcttagattatcatataatatacaaagtttatgggtcttatttcttctgtcttttagcagagggctcaatatcagtggcactgacgaactcttcttcgtattcttgtccatcatccgaatacgacgcttcatcttcgtagtc |
| T_LEU2_-T_NFS1_ | aaagattctctttttttatgatatttgtacataaactttataaatgaaattcataatagaaacgacacgaaattacaaaatggaatatgttcatagggtagacgaaactatatacgcaatctacatacatttatcaagaaggagaaaaaggaggatgtaaaggaatacaggtaagcaaattgatactaatggctcaacgtgataaggaaaaagaattgcactttaacattaatattgacaaggaggagggcaccacacaaaaagttaggtgtaacagaaaatcatgaaactatgattcctaatttatatattggaggattttctctaaaaaaaaaaaaatacaacaaataaaaaacac |
| T_DSF1_-T_HXT13_ | ttttaaaggtgaactgatctacgcgccctcgatagtaatgactaaatatcttgggtagagtatatataatgtcgtatttttgtatattgttttatttagacaaatagtaacgtgttatgttccttcaatcgcatctttcatgatctttaatcgatcgtcaaatggatccatttagagtttctcatcaccatccccatatcatttcactccaccccgctttacgtaaaaaaaaaaaaaaaaattgaataaatgactaagaattagacacaattttgtcttaatgaatgctttttacttatgacacatgccagtttgtacatatgttgatcttcatagctccgataatcttcataaattcgtgacaaattaaaattacacattattatgtaaactataatatacaatgttgcctatcaagacaaacatatgcactctatga |
| T_TIM21_-T_GSC2_ | cactcatacgccatccttaaagacctggtctacgatcaaatgatttttttagtttacaatctatttttgtttctaagcaagtttatcacgcaaatacataagtatatttttactttctattcttcctagtttatatttatttcattgtaactttcttagaagctcggtcctctcgctatatagtaggatctgcaacatatttggatgtgggtgggcgttctccttcttttttagatgtaaggtccaacacgtataacaggtgatacacatagaaagacacgtggaaataacagtcatttacgaatatttaaaacctgagcaactccgtcaaatttgatcttaatcttttctggggccccatctaattcccagaaagcccttcgaattagaaaccggatgc |
| T_RRP12_-T_TAF3_ | ggtaacaagattggcaaacataataagaaaggtccaaagttcaaatctagaaaaaaattatagaagattgaaactgagcaatatggctaattacacacctggagaaaaaatcagatatgtatatataagaatattataatactgtatattaaaaatgattaaaataaagaaaaaaatgaatcgggcgtttaattgcttattatcttgaagaagcgaaagtacactatatagtaataatgtgaggttaattaaatatggatgagataatgacgaaagaaaatgcagaaatgtcgttttaaaagtaacccccataatctagtgaggttcgacg |
| T_RNA14_-T_BUB2_ | gttaacattacgttaataaataggtatatatgaatatttataccaacacatctattataataggcgaacctctgtatgtaattaagtaaaaaaaaaacgatgtgacaggatagttaaggtgcctcgtacataaataaaaacggaaatagttaattctttcaaaaatatggcaatagccaaactcattcagaaggtacaggaaacactctgtttctgtgcgtttatataaccatgcttataaaagaaagattgaacaaaatatacatgaatttatgaacggtaatcaccgttaattgttaca |
| T_ADH1_ | cattccgttggtagatacgttgttgacacttctaaataagcgaatttcttatgatttatgatttttattattaaataagttataaaaaaaataagtgtatacaaattttaaagtgactcttaggttttaaaacgaaaattcttattcttgagtaactctttcctgtaggtcaggttgctttctcaggtatagcatgaggtcgctc |
| T_CYC1_ | atccgctctaaccgaaaaggaaggagttagacaacctgaagtctaggtccctatttatttttttatagttatgttagtattaagaacgttatttatatttcaaatttttcttttttttctgtacagacgcgtgtacgcatgtaacattatactgaaaaccttgcttgagaaggttttgggacgctcgaag |
| T_TDH3_ | gtgaatttactttaaatcttgcatttaaataaattttctttttatagctttatgacttagtttcaatttatatactattttaatgacattttcgattcattgattgaaagctttgtgttttttcttgatgcgctattgcattgttcttgtctttttcgccacatgtaatatctgtagtagatacctgatacattgtggatgctgagtgaaattttagttaataatggaggcgctcttaataattttggggatattggcttttttttttaaagtttacaaatgaattttttccgccaggataacgattctgaagttactcttagcgttcctatcggtacagccatcaaatcatgcctataaatcatgcctatatttgcgtgcagtcagtatcatctacatgaaaaaaactcccgcaatttcttatagaatacgttgaaaattaaatgtacgcgccaagataagataacatatatctagatgcagtaatatacacagattcccgcggacgtgggaaggaaaaaattagataacaaaatctgagtgatatggaaattccgctgtatagctcatatctttccct |
| Ty1Cons1 up LTR | tccgcgctgagggtttaatggcgcgccgcggccgcccgcggtgttggaataaaaatccactatcgtctatcaactaatagttatattatcaatatattatcatatacggtgttaagatgatgacataagttatgagaagctgtcatcgaagttagaggaagctgaagtgcaaggattgataatgtaataggatcaatgaatataaacatat |
| Ty1Cons1 dn LTR | aaaacggaatgaggaataatcgtaatattagtatgtagaaatatagattccattttgaggattcctatatcctcgaggagaacttctagtgtatattctgtatacctaatattatagcctttatcaacaatggaatcccaacaattatctaattacccacaaatttctcaagatctgcggccgcactcagacctgaagtgaagttcctatact |
| Ty1Cons1 up LTR | ccgcgctgagggtttaatggcgcgccgcggccgcccgcggtgttggaataaaaatcaactatcatctactaactagtatttacgttactagtatattatcatatacggtgttagaagatgacgcaaatgatgagaaatagtcatctaaattagtggaagctgaaacgcaaggattgataatgtaataggatcaatgaatattaacata |
| Ty1Cons2 dn LTR | taaaacggaatgatgaataatatttatagaattgtgtagaattgcagattcccttttatggattcctaaatcctcgaggagaacttctagtatattctgtatacctaatattatagcctttatcaacaatggaatcccaacaattatctcaaaattcacatatttctcaagatctgcggccgcactcagacctgaagtgaagttcctatac |
| Ty2Cons up LTR | gtgtccgcgctgagggtttaatggcgcgccgcggccgcccgcggtgttggaataaaaatcaactatcatctactaactagtatttacgttactagtatattatcatatacggtgttagaagatgacgcaaatgatgagaaatagtcatctaaattagtggaagctgaaacgcaaggattgataatgtaataggatcaatgaatattaac |
| Ty2Cons dn LTR | atataaaatgatgataataatatttatagaattgtgtagaattgcagattcccttttatggattcctaaatcctgaggagaacttctagtatattctacatacctaatattattgccttattaaaaatggaatcccaacaattacatcaaaatccacattctcagatctgcggccgcactcagacctgaagtgaagttcctatac |
| Ty3Cons up LTR | gtccgcgctgagggtttaatggcgcgccgcggccgcccgcggtgttgtatctcaaaatgagatatgtcagtatgacaatacgtcatcctgaacgttcataaaacacatatgaaacaaccttataacaaaacgaacaacatgagacaaaacccgtccttccctagctgaactacccaaaagtataaatgcctgaacaattagtttagatccga |
| Ty3Cons dn LTR | gattccgcgcttccaccacttagtatgattcatattttatataatatataagataagtaacattccgtgaattaatctgataaactgttttgacaactggttacttccctaagactgtttatattaggattgtcaagacactccggtattactcgagcccgtaatacaacaagatctgcggccgcactcagacctgaagtgaagttcctatac |
| Ty4Cons up LTR | gaacttctgaagtggggatttaaatgcggccgcgctgagggtttaatggcgcgccgcggccgcccgcggtgttggaacgagagtaattaatagtgacatgagttgctatggtaacaatctaatgcttacatcgtatattaatgtacaactcgtatacgtttaagtgtgattgcgcctattgcagaaggaatgttaaacgagaagctcagacaatactgaagctgtgttaaagacctattagttgaacatgttatgctagcattaagtcctcagcgagctcgcatggaatgcgtgcgat |
| Ty4Cons dn LTR | taggtgatatcagatccactagtggcctatgcacccaattcgccctatagtgagtcgtattacgcgcgctcactggccgtcgttttacaacgtcgtgactgggaaaaccctggcgttacccctacaggactagtgctgaggcattaattgatcaggtaggtacatatatgaggaatatgagtcgtcacatcaatgtatagtaactaccggaatcactattatattggtcatgattaatatgaccaatcggcgtgtgttttatatacctctcttatttagtataagaagatcagtactcacttcttcattaatactaatttttaacctctaattatcaacaagatctgcggccgcggccgcaaatttaaataaaatgaagtgaagttcctatactttctaga |
| 26s rDNA up | atgagagtagcaaacgtaagtctaaaggttgttttatagtagttaggatgtagaaaatgtattccgataggccattttacatttggagggacggttgaaagtggacagaggaaaaggtgcggaaatggctgattttgattgtttatgttttgtgtgatgattttacatttttgcatagtattaggtagtcagatgaaagatgaatagacataggagtaagaaaacatagaatagttaccgttattggtaggagtgtggtggggtggtatagtccgcattgggatgttactttcctgttatggcatggatttccctttagggtctctgaagcgtatttccgtcaccgaaaaaggcagaaaaagggaaactgaagggaggatagtagtaaagtttgaatggtggtagtgtaatgtatgatatccgttggttttggtttcggttgtgaaaagttttttggtatgatattttgcaagtagcatatatttcttgtgtgagaaaggtatattttgtatgttttgtatgttcccgcgcgtttccgtattttccgcttccgcttccgcagtaaaaaatagtgaggaactgggttacccggggcacctgtcactttggaaaaaaaatatacgctaagatttttggagaatagcttaaattgaagtttttctcggcgagaaatacgtagttaaggcagagcgac |
| 26s rDNA dn | agagggcaaaagaaaataaaagtaagattttagtttgtaatgggagggggggtttagtcatggagtacaagtgtgaggaaaagtagttgggaggtacttcatgcgaaagcagttgaagacaagttcgaaaagagtttggaaacgaattcgagtaggcttgtcgttcgttatgtttttgtaaatggcctcgtcaaacggtggagagagtcgctaggtgatcgtcagatctgcctagtctctatacagcgtgtttaattgacatgggttgatgcgtattgagagatacaatttgggaagaaattcccagagtgtgtttcttttgcgtttaacctgaacagtctcatcgtgggcatcttgcgattccattggtgagcagcgaaggatttggtggattactagctaatagcaatctatttcaaagaattcaaacttgggggaatgccttgttgaatagccggtcgcaagactgtgattcttcaagtgtaacctcctctcaaatcagcgatatcaaacgtaccattccgtgaaacaccggggtatctgtttggtggaacctgattagaggaa |

## Table S4 Primers used in this manuscript

| **Primers** | **Sequences (5′-3′)** |
| --- | --- |
| Tvector-F | gttatccgcttttccataggctccgcccccctgacgagcatcaca |
| Tvector-R | aaccctggcgtttgtttatttttctaaatacattcaaatatgtatcc |
| M13-F | aataaacaaacgccagggttttcccagtcacgacgttgtaaaacg |
| M13-R | cctatggaaaagcggataacaatttcacacaggaaacagctatgac |
| LUE2-F | tccagagattgagctcgctgtgaagatcccagcaaaggcttacaaa |
| LEU2-R | aattcacttacaaatgaataacgaaatgagacaaagaagagaacc |
| 2TTDH3-F | tcatttgtaagtgaatttactttaaatcttgcatttaaataaattttc |
| 2TTDH3-R | ttcgacgattagggaaagatatgagctatacagcggaatttcca |
| pT2-F | atctttccctaatcgtcgaacggcaggcgtgcaaacttggcgtaatc |
| pT2-R | cagcgagctcaatctctggaagatccgcgcgtaccgagttctaattc |
| URA3-F | tccagagattttttatttaggttctatcgaggagaaaaagcgaca |
| URA3-R | aattcacttacaaatgaataacgaaatgagacaaagaagagaacc |
| 3TTDH3-F | tcatttgtaagtgaatttactttaaatcttgcatttaaataaattttc |
| 3TTDH3-R | ttcgacgattagggaaagatatgagctatacagcggaatttcca |
| pT3-F | atctttccctaatcgtcgaacggcaggcgtgcaaacttggcgtaatc |
| PT3-R | ctaaataaaaaatctctggaagatccgcgcgtaccgagttctaattc |
| ADE6-F | tccagagattctgaacgtatcgagactcggttgtgtcgttatgc |
| ADE6-R | tcaccgacattgaacttgacttcttttgttatggacctgggagg |
| TRP1-F | gtcaagttcaatgtcggtgataaactttacaggaagtagtggacc |
| TRP1-R | ttttacaagccttctttgcattttttacgaaattagctattttattac |
| pT1-F | tgcaaagaaggcttgtaaaaattggttctcttctttgtctcatttcg |
| pT1-R | atacgttcagaatctctggaagatccgcgcgtaccgagttctaattc |
| ZWF1-F | tccagagattgccgtcgaaaaggatctcgtctctgttgggagcacct |
| ZWF1-R | gttctgccatcttgccttatgtggttttctattctattggatttac |
| HIS5-F | ataaggcaagatggcagaaccagcccaaaaaaagcaaaaacaaac |
| HIS5-R | ttttacaagccatcaaaacacctttggttgagggaacgtcattgg |
| pT5-F | tgttttgatggcttgtaaaaattggttctcttctttgtctcatttcg |
| pT5-R | tttcgacggcaatctctggaagatccgcgcgtaccgagttctaattc |
| ARO7-F | tccagagatttggattacatttgattcagtcatacacgaattatgg |
| ARO7-R | tggtacccatatcttataccaattttatgcaggatgctgagtgctatttg |
| nat-F | ggtataagatatgggtaccactcttgacgacacggcttaccggtac |
| nat-R | ttttacaagcggggcagggcatgctcatgtagagcgcctgctcgc |
| pT6-F | gccctgccccgcttgtaaaaattggttctcttctttgtctcatttcg |
| pT6-R | atgtaatccaaatctctggaagatccgcgcgtaccgagttctaattc |
| PYC1-F | tccagagattcgtatccaagccgaaacggcgctcgcctcatcccc |
| PYC1-R | ttttacccattgtattctgatagtatgtgtttgtgtatgttaaag |
| hph-F | tcagaatacaatgggtaaaaagcctgaactcaccgcgacgtctgtc |
| hph-R | ttttacaagcttcctttgccctcggacgagtgctggggcgtcgg |
| pT7-F | ggcaaaggaagcttgtaaaaattggttctcttctttgtctcatttcg |
| pT7-R | cttggatacgaatctctggaagatccgcgcgtaccgagttctaattc |
| ADE3-F | tccagagattacgtgagctaaagcacagattgttggaaaagcaag |
| ADE3-R | ccttacccatgatttcttatttgacttgtaaagttgtgttcgtctc |
| Kan-F | ataagaaatcatgggtaaggaaaagactcacgtttcgaggccgcg |
| Kan-R | ttttacaagcgaaaaactcatcgagcatcaaatgaaactgcaatttattc |
| pT8-F | tgagtttttcgcttgtaaaaattggttctcttctttgtctcatttcg |
| pT8-R | tagctcacgtaatctctggaagatccgcgcgtaccgagttctaattc |
| YEF3-F | tccagagattaataagcgccactatcagggaatagcaactttccc |
| YEF3-R | ggctacccatcttttaatgttatcgatggattcgaac |
| pat-F | acattaaaagatgggtagcccagaacgacgcccggtcgagatccg |
| pat-R | ttttacaagcgatctgtgtgacgggccggacggggcggggcgggg |
| pT9-F | cacacagatcgcttgtaaaaattggttctcttctttgtctcatttcg |
| pT9-R | ggcgcttattaatctctggaagatccgcgcgtaccgagttctaattc |
| ERG1-F | tccagagattgtgaatggtatgaacatggacatgagcgtggttcag |
| ERG1-R | ggtcggccatgacccttttctcgatatgtttttctgtgatttttttttttc |
| ble-F | gaaaagggtcatggccgaccaagcgacgcccaacctgccatcacg |
| ble-R | ttttacaagctgagatgcctgcaagcaattcgttctgtatcaggc |
| pT0-F | aggcatctcagcttgtaaaaattggttctcttctttgtctcatttcg |
| pT0-R | taccattcacaatctctggaagatccgcgcgtaccgagttctaattc |
| Ty11up-F | ttccagagattccgcgctgagggtttaatggcgcgccgcggccgcc |
| Ty11up-R | aaaacacttgatatgtttatattcattgatcctattacattatcaa |
| TT11up-F | ataaacatatcaagtgtttttgacgaaagattatcacttggaaaca |
| TT11up-R | agctggcaaactccagcgaagctcctgctttattccaatttgatttg |
| 11E-F | ttcgctggagtttgccagcttactatccttcttgaaaatatgcac |
| 11E-R | ttgatactctttatttgtatagttcatccatgccatgtgtaatcc |
| TT11dn-F | atacaaataaagagtatcaagaatttaaaatgaaacatctcaaaaga |
| TT11dn-R | attccgttttttgttataacttaaatacctctatttattacatag |
| Ty11dn-F | gttataacaaaaaacggaatgaggaataatcgtaatattagtatg |
| Ty11dn-R | gttcgacgatagtataggaacttcacttcaggtctgagtgcggcc |
| pcT11E-F | ttcctatactatcgtcgaacggcaggcgtgcaaacttggcgtaatc |
| pcT11E-R | tcagcgcggaatctctggaagatccgcgcgtaccgagttctaattc |
| Ty12up-F | ttccagagatccgcgctgagggtttaatggcgcgccgcggccgcc |
| Ty12up-R | aagagaagcttatgttaatattcattgatcctattacattatcaa |
| TT12up-F | tattaacataagcttctctttacgttccaatgactggtaaagttac |
| TT12up-R | agctggcaaagactacgaagatgaagcgtcgtattcggatgatgg |
| 12E-F | cttcgtagtctttgccagcttactatccttcttgaaaatatgcac |
| 12E-R | gagaatctttttatttgtatagttcatccatgccatgtgtaatcc |
| TT12dn-F | atacaaataaaaagattctctttttttatgatatttgtacataaa |
| TT12dn-R | ttccgttttagtgttttttatttgttgtatttttttttttttaga |
| Ty12dn-F | taaaaaacactaaaacggaatgatgaataatatttatagaattgt |
| Ty12dn-R | gttcgacgatgtataggaacttcacttcaggtctgagtgcggccg |
| pcT12E-F | gttcctatacatcgtcgaacggcaggcgtgcaaacttggcgtaatc |
| pcT12E-R | ctcagcgcggatctctggaagatccgcgcgtaccgagttctaattc |
| Ty2up-F | ttccagagatgtgtccgcgctgagggtttaatggcgcgccgcggc |
| Ty2up-R | ggattgataatgtaataggatcaatgaatattaacttttaaaggt |
| TT2up-F | gaatattaacttttaaaggtgaactgatctacgcgccctcgatag |
| TT2up-R | agctggcaaatcatagagtgcatatgtttgtcttgataggcaaca |
| 2E-F | cactctatgatttgccagcttactatccttcttgaaaatatgcact |
| 2E-R | cgtatgagtgttatttgtatagttcatccatgccatgtgtaatcc |
| TT2dn-F | atacaaataacactcatacgccatccttaaagacctggtctacga |
| TT2dn-R | cattttatatgcatccggtttctaattcgaagggctttctgggaa |
| Ty2dn-F | aaccggatgcatataaaatgatgataataatatttatagaattgt |
| Ty2dn-R | gttcgacgatgtataggaacttcacttcaggtctgagtgcggccg |
| pcT2E-F | gttcctatacatcgtcgaacggcaggcgtgcaaacttggcgtaatca |
| pcT2E-R | gcgcggacacatctctggaagatccgcgcgtaccgagttctaattc |
| Ty3up-F | ttccagagatgtccgcgctgagggtttaatggcgcgccgcggccg |
| Ty3up-R | tcttgttacctcggatctaaactaattgttcaggcatttatacttttg |
| TT3up-F | ttagatccgaggtaacaagattggcaaacataataagaaaggtcca |
| TT3up-R | agctggcaaacgtcgaacctcactagattatgggggttactttta |
| 3E-F | aggttcgacgtttgccagcttactatccttcttgaaaatatgcact |
| 3E-R | taatgttaacttatttgtatagttcatccatgccatgtgtaatcc |
| TT3dn-F | atacaaataagttaacattacgttaataaataggtatatatgaat |
| TT3dn-R | gcgcggaatctgtaacaattaacggtgattaccgttcataaattc |
| Ty3dn-F | aattgttacagattccgcgcttccaccacttagtatgattcatat |
| Ty3dn-R | gttcgacgatgtataggaacttcacttcaggtctgagtgcggccg |
| pcT3E-F | gttcctatacatcgtcgaacggcaggcgtgcaaacttggcgtaatca |
| pcT3E-R | cagcgcggacatctctggaagatccgcgcgtaccgagttctaattc |
| Ty4up-F | ttccagagatgaacttctgaagtggggatttaaatgcggccgcgct |
| Ty4up-R | gaggtcgctcatcgcacgcattccatgcgagctcgctgaggactta |
| TT4up-F | tgcgtgcgatgagcgacctcatgctatacctgagaaagcaacctgac |
| TT4up-R | agctggcaaacattccgttggtagatacgttgttgacacttctaa |
| 4E-F | caacggaatgtttgccagcttactatccttcttgaaaatatgcact |
| 4E-R | tagagcggatttatttgtatagttcatccatgccatgtgtaatcc |
| TT4dn-F | atacaaataaatccgctctaaccgaaaaggaaggagttagacaac |
| TT4dn-R | atatcacctacttcgagcgtcccaaaaccttctcaagcaaggttt |
| Ty4dn-F | acgctcgaagtaggtgatatcagatccactagtggcctatgcacc |
| Ty4dn-R | ttcgacgattctagaaagtataggaacttcacttcattttatttaaatttg |
| pcT4E-F | actttctagaatcgtcgaacggcaggcgtgcaaacttggcgtaatc |
| pcT4E-R | agttcatctctggaagatccgcgcgtaccgagttctaattc |
| mpT111-F | gttataacaattacaaatgaataacgaaatgagacaaagaagaga |
| mpT111-R | attccgttttctgaacgtatcgagactcggttgtgtcgttatgcta |
| pcT111-F | atacgttcagaaaacggaatgaggaataatcgtaatattagtatg |
| pcT111-R | tcatttgtaattgttataacttaaatacctctatttattacatag |
| mpT121-F | taaaaaacacttacaaatgaataacgaaatgagacaaagaagaga |
| mpT121-R | ttccgttttactgaacgtatcgagactcggttgtgtcgttatgcta |
| pcT121-F | atacgttcagtaaaacggaatgatgaataatatttatagaattgtgt |
| pcT121-R | tcatttgtaagtgttttttatttgttgtatttttttttttttaga |
| mpT21-F | aaccggatgcttacaaatgaataacgaaatgagacaaagaagagaa |
| mpT21-R | cattttatatctgaacgtatcgagactcggttgtgtcgttatgct |
| pcT21-F | atacgttcagatataaaatgatgataataatatttatagaattgtg |
| pcT21-R | tcatttgtaagcatccggtttctaattcgaagggctttctgggaa |
| mpT31-F | aattgttacattacaaatgaataacgaaatgagacaaagaagaga |
| mpT31-R | gcgcggaatcctgaacgtatcgagactcggttgtgtcgttatgcta |
| pcT31-F | atacgttcaggattccgcgcttccaccacttagtatgattcatattttata |
| pcT31-R | tcatttgtaatgtaacaattaacggtgattaccgttcataaattc |
| mpT112-R | attccgttttgagctcgctgtgaagatcccagcaaaggcttacaa |
| pcT112-F | cagcgagctcaaaacggaatgaggaataatcgtaatattagtatg |
| mpT122-R | ttccgttttagagctcgctgtgaagatcccagcaaaggcttacaa |
| pcT122-F | cagcgagctctaaaacggaatgatgaataatatttatagaattgtg |
| mpT22-R | cattttatatgagctcgctgtgaagatcccagcaaaggcttacaa |
| pcT22-F | cagcgagctcatataaaatgatgataataatatttatagaattgtgt |
| mpT32-R | gcgcggaatcgagctcgctgtgaagatcccagcaaaggcttacaa |
| pcT32-F | cagcgagctcgattccgcgcttccaccacttagtatgattcatattttata |
| mpT42-R | atatcacctagagctcgctgtgaagatcccagcaaaggcttacaa |
| pcT42-F | cagcgagctctaggtgatatcagatccactagtggcctatgcaccc |
| mpT113-R | attccgttttttttatttaggttctatcgaggagaaaaagcgaca |
| pcT113-F | ctaaataaaaaaaacggaatgaggaataatcgtaatattagtatg |
| mpT123-R | ttccgttttattttatttaggttctatcgaggagaaaaagcgaca |
| pcT123-F | ctaaataaaataaaacggaatgatgaataatatttatagaattgtgt |
| mpT23-R | cattttatatttttatttaggttctatcgaggagaaaaagcgaca |
| pcT23-F | ctaaataaaaatataaaatgatgataataatatttatagaattgtgt |
| mpT33-R | gcgcggaatcttttatttaggttctatcgaggagaaaaagcgaca |
| pcT33-F | ctaaataaaagattccgcgcttccaccacttagtatgattcatattttata |
| mpT43-R | atatcacctattttatttaggttctatcgaggagaaaaagcgaca |
| pcT43-F | ctaaataaaataggtgatatcagatccactagtggcctatgcacc |
| mpT115-R | attccgttttgccgtcgaaaaggatctcgtctctgttgggagcac |
| pcT115-F | tttcgacggcaaaacggaatgaggaataatcgtaatattagtatg |
| mpT125-R | ttccgttttagccgtcgaaaaggatctcgtctctgttgggagcac |
| pcT125-F | tttcgacggctaaaacggaatgatgaataatatttatagaattgtg |
| mpT25-R | cattttatatgccgtcgaaaaggatctcgtctctgttgggagca |
| pcT25-F | tttcgacggcatataaaatgatgataataatatttatagaattgtg |
| mpT35-R | gcgcggaatcgccgtcgaaaaggatctcgtctctgttgggagcac |
| pcT35-F | tttcgacggcgattccgcgcttccaccacttagtatgattcatattttatataa |
| mpT45-R | atatcacctagccgtcgaaaaggatctcgtctctgttgggagcac |
| pcT45-F | tttcgacggctaggtgatatcagatccactagtggcctatgcacc |
| mpT116-R | attccgtttttggattacatttgattcagtcatacacgaattatg |
| pcT116-F | atgtaatccaaaaacggaatgaggaataatcgtaatattagtatg |
| mpT126-R | ttccgttttatggattacatttgattcagtcatacacgaattatg |
| pcT126-F | atgtaatccataaaacggaatgatgaataatatttatagaattgtg |
| mpT26-R | cattttatattggattacatttgattcagtcatacacgaattatg |
| pcT26-F | atgtaatccaatataaaatgatgataataatatttatagaattgtg |
| mpT36-R | gcgcggaatctggattacatttgattcagtcatacacgaattatg |
| pcT36-F | atgtaatccagattccgcgcttccaccacttagtatgattcatattttatata |
| mpT46-R | atatcacctatggattacatttgattcagtcatacacgaattatg |
| pcT46-F | aaatgtaatccataggtgatatcagatccactagtggcctatgcaccc |
| mpT117-R | attccgttttcgtatccaagccgaaacggcgctcgcctcatcccc |
| pcT117-F | cttggatacgaaaacggaatgaggaataatcgtaatattagtatg |
| mpT127-R | ttccgttttacgtatccaagccgaaacggcgctcgcctcatcccc |
| pcT127-F | cttggatacgtaaaacggaatgatgaataatatttatagaattgtg |
| mpT27-R | cattttatatcgtatccaagccgaaacggcgctcgcctcatccc |
| pcT27-F | cttggatacgatataaaatgatgataataatatttatagaattgtg |
| mpT37-R | gcgcggaatccgtatccaagccgaaacggcgctcgcctcatcccc |
| pcT37-F | cttggatacggattccgcgcttccaccacttagtatgattcatattttatataata |
| mpT47-R | atatcacctacgtatccaagccgaaacggcgctcgcctcatcccc |
| pcT47-F | cttggatacgtaggtgatatcagatccactagtggcctatgcacc |
| mpT118-R | attccgttttacgtgagctaaagcacagattgttggaaaagcaag |
| pcT118-F | tttagctcacgtaaaacggaatgaggaataatcgtaatattagtatg |
| mpT128-R | ttccgttttaacgtgagctaaagcacagattgttggaaaagcaag |
| pcT128-F | tagctcacgttaaaacggaatgatgaataatatttatagaattgtg |
| mpT28-R | cattttatatacgtgagctaaagcacagattgttggaaaagcaag |
| pcT28-F | tagctcacgtatataaaatgatgataataatatttatagaattgtg |
| mpT38-R | aagcgcggaatcacgtgagctaaagcacagattgttggaaaagcaag |
| pcT38-F | agctcacgtgattccgcgcttccaccacttagtatgattcatattttatataata |
| mpT48-R | atatcacctaacgtgagctaaagcacagattgttggaaaagcaagc |
| pcT48-F | tagctcacgttaggtgatatcagatccactagtggcctatgcacc |
| mpT119-R | attccgttttaataagcgccactatcagggaatagcaactttccc |
| pcT119-F | ggcgcttattaaaacggaatgaggaataatcgtaatattagtatg |
| mpT129-R | ttccgttttaaataagcgccactatcagggaatagcaactttccc |
| pcT129-F | ggcgcttatttaaaacggaatgatgaataatatttatagaattgtg |
| mpT29-R | cattttatataataagcgccactatcagggaatagcaactttccc |
| pcT29-F | ggcgcttattatataaaatgatgataataatatttatagaattgtg |
| mpT39-R | gcgcggaatcaataagcgccactatcagggaatagcaactttccc |
| pcT39-F | ggcgcttattgattccgcgcttccaccacttagtatgattcatattttatataata |
| mpT49-R | atatcacctaaataagcgccactatcagggaatagcaactttccc |
| pcT49-F | ggcgcttatttaggtgatatcagatccactagtggcctatgcacc |
| mpT110-R | attccgttttgtgaatggtatgaacatggacatgagcgtggttca |
| pcT110-F | taccattcacaaaacggaatgaggaataatcgtaatattagtatgt |
| mpT120-R | ttccgttttagtgaatggtatgaacatggacatgagcgtggttca |
| pcT120-F | taccattcactaaaacggaatgatgaataatatttatagaattgtgt |
| mpT20-R | cattttatatgtgaatggtatgaacatggacatgagcgtggttca |
| pcT20-F | taccattcacatataaaatgatgataataatatttatagaattgtg |
| mpT30-R | gcgcggaatcgtgaatggtatgaacatggacatgagcgtggttca |
| pcT30-F | taccattcacgattccgcgcttccaccacttagtatgattcatattttatataata |
| mpT40-R | atatcacctagtgaatggtatgaacatggacatgagcgtggttca |
| pcT40-F | taccattcactaggtgatatcagatccactagtggcctatgcacc |
| pT1A-F | gtcaagttcaaagtcggtgataaactttacaggaagtagtgg |
| pT1A-R | tcaccgactttgaacttgacttcttttgttatggacctgggagg |
| pT1G-F | gtcaagttcagtgtcggtgataaactttacaggaagtagtgg |
| pT1G-R | tcaccgacactgaacttgacttcttttgttatggacctgggagg |
| pT2G-F | agattataccgtgtctaagaatatcgttgtcctaccgggtgatcacg |
| pT2G-R | tcttagacacggtataatctgtgtagtgtgggatactttttacttc |
| pT2A-F | agattataccaagtctaagaatatcgttgtcctaccgggtgatcacg |
| pT2A-R | tcttagacttggtataatctgtgtagtgtgggatactttttacttc |
| rDNAup-F | tccagagattatgagagtagcaaacgtaagtctaaaggttgt |
| rDNAup-R | tttgccctctgtcgctctgccttaactacgtatttctcgccgagaa |
| rDNAdn-F | aggcagagcgacagagggcaaaagaaaataaaagtaagattttagtttg |
| rDNAdn-R | ttcgacgattttcctctaatcaggttccaccaaacagataccccg |
| rTRP1-F | gcagagcgaccaagtgtttttgacgaaagattatcacttggaaac |
| rTRP1-R | tttgccctctctgaacgtatcgagactcggttgtgtcgttatgc |
| rLEU2-F | gcagagcgacagcttctctttacgttccaatgactggtaaagttac |
| rLEU2-R | tttgccctctgagctcgctgtgaagatcccagcaaaggcttac |
| rURA3-F | gcagagcgacttttaaaggtgaactgatctacgcgccctcgatag |
| rURA3-R | tttgccctctttttatttaggttctatcgaggagaaaaagcgaca |
| rTRP1AAG-F | gcagagcgaccaagtgtttttgacgaaagattatcacttggaaac |
| rTRP1AAG-R | tttgccctctctgaacgtatcgagactcggttgtgtcgttatgc |
| rHIS5-F | gcagagcgacgagcgacctcatgctatacctgagaaagcaacc |
| rHIS5-R | tttgccctctgccgtcgaaaaggatctcgtctctgttgggagcac |
| rnat-F | gcagagcgacggtaacaagattggcaaacataataagaaaggtcc |
| rnat-R | tttgccctcttggattacatttgattcagtcatacacgaattatgg |
| rhph-F | gcagagcgacggtaacaagattggcaaacataataagaaaggtcc |
| rhph-R | tttgccctctcgtatccaagccgaaacggcgctcgcctcatcccc |
| RTRP1-F | atacgttcagagagggcaaaagaaaataaaagtaagattttagtttgt |
| RTRP1-R | aaaacacttggtcgctctgccttaactacgtatttctcgccgag |
| RLEU2-F | cagcgagctcagagggcaaaagaaaataaaagtaagattttagt |
| RLEU2-R | aagagaagctgtcgctctgccttaactacgtatttctcgccgag |
| RURA3-F | ctaaataaaaagagggcaaaagaaaataaaagtaagattttag |
| RURA3-R | acctttaaaagtcgctctgccttaactacgtatttctcgccgag |
| RTRP1AAG-F | atacgttcagagagggcaaaagaaaataaaagtaagattttagtttgt |
| RTRP1AAG-R | aaaacacttggtcgctctgccttaactacgtatttctcgccgag |
| RHIS5-F | tttcgacggcagagggcaaaagaaaataaaagtaagattttagtttg |
| RHIS5-R | gaggtcgctcgtcgctctgccttaactacgtatttctcgccgag |
| Rnat-F | atgtaatccaagagggcaaaagaaaataaaagtaagattttag |
| Rnat-R | tcttgttaccgtcgctctgccttaactacgtatttctcgccgag |
| Rhph-F | cttggatacgagagggcaaaagaaaataaaagtaagattttag |
| Rhph-R | tcttgttaccgtcgctctgccttaactacgtatttctcgccgag |
| PINO1-F | tccagagattgaagacgatgaggccggtgccgatgtgcccttgatg |
| PINO-R | gttgtgtcattgttacttctttttcactggaaaaaaaagggaatg |
| aroL-F | agaagtaacaatgacacaacctctttttctgatcgggcctcggg |
| aroL-R | ttcgacgatttcaacaattgatcgtctgtgccagggcgctgcga |
| pT100-F | caattgttgaaatcgtcgaacggcaggcgtgcaaacttggcgtaatc |
| pT100-R | catcgtcttcaatctctggaagatccgcgcgtaccgagttctaattc |
| LL-F | gaaatagcattccgttggtagatacgttgttgacacttctaaataagcg |
| LL-R | attgttgacattcatccgctctaaccgaaaaggaaggagttaga |
| LL47f-F | cggaatgctatttcttgttaacttctcttctttgtctgacagc |
| LL47f-R | agctggcaaattactcttccaaccttcttagcaagtattccacctc |
| LLTAL-F | tggaagagtaatttgccagcttactatccttcttgaaaatatgcac |
| LLTAL-R | catcgtcttcttaattgttaatcaaatgatccttaaccttttgta |
| LLaroL-F | taacaattaagaagacgatgaggccggtgccgatgtgcccttgatg |
| LLaroL-R | gatgaatgtcaacaattgatcgtctgtgccagggcgctgcgaatttc |
| PFBA1-F | tccagagattataacaatactgacagtactaaataattgcctac |
| PFBA1-R | attgatccattttgaatatgtattacttggttatggttatatatg |
| PAL-F | catattcaaaatggatcaatataccaatggacattccactagtaa |
| PAL-R | ttcgacgattttatgaaattggaagtggctttccgtcccacatcttc |
| pT101-F | aatttcataaaatcgtcgaacggcaggcgtgcaaacttggcgtaa |
| pT101-R | gtattgttataatctctggaagatccgcgcgtaccgagttctaattc |
| PTDH1-F | tccagagattgaaaccacaccgtggggccttgttgcgctaggaa |
| PTDH1-R | gaagatccattttgttttgtgtgtaaatttagtgaagtactg |
| C4H-F | acaaaacaaaatggatcttctccttttggagaaagctcttgtagg |
| C4H-R | ttcgacgatttcacaatgatcttggtttagcaacaacggtggaatg |
| pT102-F | atcattgtgaaatcgtcgaacggcaggcgtgcaaacttggcgtaatc |
| pT102-R | gtgtggtttcaatctctggaagatccgcgcgtaccgagttctaattc |
| LHL-F | caattgttgatttgccagcttactatccttcttgaaaatatgcactctatatcttttag |
| LHL-R | caagaaatagtcatagagtgcatatgtttgtcttgataggcaacattg |
| LHL47f-F | cactctatgactatttcttgttaacttctcttctttgtctgacagc |
| LHL-47f-R | gtattgttatttactcttccaaccttcttagcaagtattccacctc |
| LLPAL-F | ggaagagtaaataacaatactgacagtactaaataattgcctacttg |
| LLPAL-R | gtgtggtttcttatgaaattggaagtggctttccgtcccacatcttc |
| LLC4H-F | aatttcataagaaaccacaccgtggggccttgttgcgctaggaatagg |
| LLC4H-R | catcgtcttctcacaatgatcttggtttagcaacaacggtggaatg |
| LLaroL-F | atcattgtgagaagacgatgaggccggtgccgatgtgcccttgatgg |
| LLaroL-R | agctggcaaatcaacaattgatcgtctgtgccagggcgctgcgaatttc |
| tax-F | caacggaatgctaagcaaatatattctcgattggcttctctttctcc |
| tax-R | cgatcgcacgttataaaccttgactgctttcatacaaatggggag |
| P5m4-F | aggtttataacgtgcgatcgcgtgcattcatccgctctaaccgaaaagg |
| P5m4-R | atttgcttagcattccgttggtagatacgttgttgacacttctaaataagc |
| LHL-2F | cactctatgactatttcttgttaacttctcttctttgtctgacagc |
| LHL-2R | cgtatgagtgtcaacaattgatcgtctgtgccagggcgctgcgaatttc |
| pcT23-2F | caattgttgacactcatacgccatccttaaagacctggtctacgatcaaatg |
| pcT23-2R | caagaaatagtcatagagtgcatatgtttgtcttgataggcaacattg |
| P03-2F | aggttcgacgttaggtagccacactatgcagaaccaccgtttccacc |
| P03-2R | taatgttaacttacttcggcaggtcgccgctcgcaatacgagcgcg |
| pcT32G-2F | gccgaagtaagttaacattacgttaataaataggtatatatgaatatttatacc |
| pcT32G-2R | ggctacctaacgtcgaacctcactagattatgggggttacttttaaaacg |
| P5m4-2F | caacggaatgctaagcaaatatattctcgattggcttctctttctcc |
| P5m4-2R | tagagcggatttataaaccttgactgctttcatacaaatggggagc |
| pcT41A-2F | aggtttataaatccgctctaaccgaaaaggaaggagttagacaacctgaag |
| pcT41A-2R | atttgcttagcattccgttggtagatacgttgttgacacttctaaataagcg |
| YFP-F | cactctatgatttgccagcttactatccttcttgaaaatatgcactc |
| YFP-R | cgtatgagtgttacaagtaagtctttctacaatcaacagctctaac |
| pcT23E-F | ttacttgtaacactcatacgccatccttaaagacctggtctacgatc |
| pcT23E-R | agctggcaaatcatagagtgcatatgtttgtcttgataggcaacattg |
| KOK-F | aggttcgacgtttgccagcttactatccttcttgaaaatatgcactc |
| KOK-R | taatgttaacttaagaatgggcaacagcatcttcaacttgttcag |
| pcT37E-F | ccattcttaagttaacattacgttaataaataggtatatatgaatatttatacc |
| pcT37E-R | agctggcaaacgtcgaacctcactagattatgggggttacttttaaaacgacatttctg |
| Kate-F | caacggaatgtttgccagcttactatccttcttgaaaatatgcactc |
| Kate-R | tagagcggattcatctgtgccccagtttgctagggaggtcgcagtatc |
| pcT44E-F | gcacagatgaatccgctctaaccgaaaaggaaggagttagacaacctg |
| pcT44E-R | agctggcaaacattccgttggtagatacgttgttgacacttctaaataagc |
| Ty11-inte-F | ccgcggtgttggaataaaaatccactatcg |
| Ty11-inte-R | agatcttgagaaatttgtgggtaattagataattg |
| Ty2-inte-F | ccgcggtgttggaataaaaatcaactatcatc |
| Ty2-inte-R | agatctgagaatgtggattttgatgtaattg |
| Ty3-inte-F | ccgcggtgttgtatctcaaaatgagatatg |
| Ty3-inte-R | agatcttgttgtattacgggctcgagtaatac |
| Ty4-inte-F | ccgcggtgttggaacgagagtaattaatag |
| Ty4-inte-R | agatcttgttgataattagaggttaaaaattag |
| rDNA-F | atgagagtagcaaacgtaagtctaaaggttg |
| rDNA-R | ttcctctaatcaggttccaccaaacagataccc |
| qACT1-F | ttattgataacggttctggtatg |
| qACT1-R | ccttggtgtcttggtctac |
| qaroL-F | gggctgtggtaaaacaacgg |
| qaroL-R | ccactcttccctttcgacga |
| qCHS-F | atcggagatactcagcgggc |
| qCHS-R | aattgacggtcacctgcgtg |
| qF3´H-F | tacccctgctactctacgcctc |
| qF3´H-R | tccaagatgaggtaagtttccga |
| qEGFP-F | ggagagggtgaaggtgatgc |
| qEGFP-R | gccgcttcatatgatctggg |
| qphiYFP-F | caatgtttcgctaagtacggtcc |
| qphiYFP-R | ttgaaaacaccgtcaccttcg |
| qmKOk-F | ggttctgcttcagtttctgctca |
| qmKOk-R | cccaatcaacagattgattttgc |
| qmKate2-F | tacggcagcaaaaccttcatc |
| qmKate2-R | gtgtcctgggtagcggtcag |

## Table S5 The qPCR data of EGFP overexpresson strains

| **strains** | **Fluorescence intensity** | **CT,EGFP** | | | **CT,ACT1** | | | **Average:CT,EGFP** | **Average: CT,ACT1** | **ΔCT** | **-ΔΔCT** |
| --- | --- | --- | --- | --- | --- | --- | --- | --- | --- | --- | --- |
| 1 | 11758 | 27.46 | 27.33 | 27.52 | 18.81 | 18.89 | 18.81 | 27.43666667 | 18.83666667 | -8.6 | 4.2067 |
| 2 | 11717 | 28.31 | 27.99 | 27.91 | 17.86 | 18.19 | 18.36 | 28.07 | 18.13666667 | -9.933333333 | 2.873366667 |
| 3 | 12407 | 31.52 | 29.95 | 29.78 | 26.43 | 26.66 | 26.39 | 30.41666667 | 26.49333333 | -3.923333333 | 8.883366667 |
| 4 | 11344 | 25.51 | 26.58 | 26.54 | 18.69 | 18.9 | 18.59 | 26.21 | 18.72666667 | -7.483333333 | 5.323366667 |
| 5 | 76769 | 15.67 | 15.69 | 15.66 | 17.59 | 17.53 | 17.45 | 15.67333333 | 17.52333333 | 1.85 | 14.6567 |
| 6 | 82816 | 14.82 | 14.68 | 14.75 | 16.59 | 16.58 | 16.69 | 14.75 | 16.62 | 1.87 | 14.6767 |
| 7 | 87238 | 15.2 | 15.26 | 15.23 | 17.3 | 17.32 | 17.07 | 15.23 | 17.23 | 2 | 14.8067 |
| 8 | 79067 | 14.77 | 14.72 | 14.71 | 16.53 | 16.48 | 16.45 | 14.73333333 | 16.48666667 | 1.753333333 | 14.56003333 |
| 9 | 143525 | 13.59 | 13.98 | 14.22 | 16.7 | 16.81 | 16.74 | 13.93 | 16.75 | 2.82 | 15.6267 |
| 10 | 116305 | 13.46 | 13.52 | 13.19 | 16.59 | 16.47 | 16.61 | 13.39 | 16.55666667 | 3.166666667 | 15.97336667 |
| 11 | 158154 | 15.35 | 15.56 | 14.72 | 18.28 | 18.22 | 18.26 | 15.21 | 18.25333333 | 3.043333333 | 15.85003333 |
| 12 | 197301 | 14.13 | 13.4 | 13.57 | 16.8 | 17.23 | 17.21 | 13.7 | 17.08 | 3.38 | 16.1867 |
| 13 | 203503 | 13.52 | 14.36 | 14.32 | 17.69 | 17.44 | 17.47 | 14.06666667 | 17.53333333 | 3.466666667 | 16.27336667 |
| 14 | 248842 | 14.5 | 13.81 | 14.84 | 17.99 | 18.92 | 18.25 | 14.38333333 | 18.38666667 | 4.003333333 | 16.81003333 |
| 15 | 260590 | 13.71 | 13.89 | 13.61 | 18.24 | 18.29 | 18.29 | 13.73666667 | 18.27333333 | 4.536666667 | 17.34336667 |
| 16 | 303764 | 14.33 | 14.58 | 14.67 | 18.25 | 17.96 | 18.41 | 14.52666667 | 18.20666667 | 3.68 | 16.4867 |
| 17 | 331231 | 13.5 | 13.54 | 14.61 | 18.51 | 18.58 | 18.34 | 13.88333333 | 18.47666667 | 4.593333333 | 17.40003333 |
| 18 | 357320 | 12.56 | 12.5 | 12.49 | 17.83 | 17.93 | 17.71 | 12.51666667 | 17.82333333 | 5.306666667 | 18.11336667 |
| 19 | 377550 | 12.64 | 12.91 | 12.82 | 17.43 | 17.67 | 17.41 | 12.79 | 17.50333333 | 4.713333333 | 17.52003333 |
| 20 | 387740 | 12.77 | 11.77 | 12.45 | 17.59 | 17.3 | 17.47 | 12.33 | 17.45333333 | 5.123333333 | 17.93003333 |
| 21 | 415981 | 16.26 | 16.58 | 16.26 | 20.47 | 20.49 | 20.29 | 16.36666667 | 20.41666667 | 4.05 | 16.8567 |
| 22 | 438188 | 12.92 | 12.92 | 12.8 | 17.75 | 17.8 | 17.86 | 12.88 | 17.80333333 | 4.923333333 | 17.73003333 |
| 23 | 468228 | 13.67 | 13.54 | 13.53 | 18.91 | 18.74 | 18.67 | 13.58 | 18.77333333 | 5.193333333 | 18.00003333 |
| 24 | 486738 | 11.84 | 11.78 | 11.67 | 17.24 | 17.3 | 17.49 | 11.76333333 | 17.34333333 | 5.58 | 18.3867 |
| 25 | 460554 | 14.26 | 13.83 | 13.83 | 19.64 | 19.61 | 19.54 | 13.97333333 | 19.59666667 | 5.623333333 | 18.43003333 |
| 26 | 472345 | 11.53 | 11.5 | 11.57 | 17.37 | 17.36 | 17.58 | 11.53333333 | 17.43666667 | 5.903333333 | 18.71003333 |
| 27 | 486738 | 12.43 | 12.24 | 12.46 | 16.3 | 16.2 | 16.17 | 12.37666667 | 16.22333333 | 3.846666667 | 16.65336667 |
| 28 | 508341 | 10.11 | 10.79 | 10.91 | 16.08 | 15.98 | 16.38 | 10.60333333 | 16.14666667 | 5.543333333 | 18.35003333 |
| C877 | 23454 | 27.5 | 27.33 | 27.99 | 17.6 | 17.56 | 17.35 | 27.60666667 | 17.50333333 | -10.10333333 | 2.703366667 |
| pY26-EGFP | 206614 | 12.57 | 12.52 | 12.4 | 17.72 | 17.59 | 17.74 | 12.49666667 | 17.68333333 | 5.186666667 | 17.99336667 |
| pY26-EGFP | 242186 | 13.43 | 13.49 | 13.46 | 18.73 | 18.77 | 18.7 | 13.46 | 18.73333333 | 5.273333333 | 18.08003333 |
| pY26-EGFP | 227175 | 11.8 | 11.65 | 11.73 | 17.16 | 16.91 | 17.14 | 11.72666667 | 17.07 | 5.343333333 | 18.15003333 |
| pY26-EGFP | 209621 | 12.92 | 13.15 | 13.91 | 18.77 | 18.79 | 18.74 | 13.32666667 | 18.76666667 | 5.44 | 18.2467 |

## Table S6 The qPCR data of protein overexpresson strains

| **Strains** | **CT,ACT1** | | | **CT,EGFP** | | | **ΔCT** | | | **-ΔΔCT** | | | **Fold Change** | | |
| --- | --- | --- | --- | --- | --- | --- | --- | --- | --- | --- | --- | --- | --- | --- | --- |
| C800E | 16.93 | 16.78 | 16.83 | 11.27 | 11.09 | 11.16 | -5.66 | -5.69 | -5.67 |  |  |  |  |  |  |
| C800Y | 16.67 | 16.65 | 16.69 | 11.52 | 11.52 | 11.47 | -5.15 | -5.13 | -5.22 |  |  |  |  |  |  |
| C80O | 16.27 | 16.35 | 16.2 | 10.79 | 10.76 | 10.79 | -5.48 | -5.59 | -5.41 |  |  |  |  |  |  |
| C800K | 16.32 | 16.26 | 16.35 | 13.24 | 13.28 | 13.39 | -3.08 | -2.98 | -2.96 |  |  |  |  |  |  |
| C811E | 16.91 | 16.79 | 16.77 | 13.22 | 13.3 | 13.2 | -3.69 | -3.49 | -3.57 | 1.97 | 2.2 | 2.1 | 3.91768119 | 4.59479342 | 4.28709385 |
| C812E | 16.11 | 15.95 | 15.95 | 12.1 | 12.31 | 12.24 | -4.01 | -3.64 | -3.71 | 1.14 | 1.49 | 1.51 | 2.203810232 | 2.808889751 | 2.848100391 |
| C802Y | 17.52 | 17.24 | 17.17 | 12.59 | 12.48 | 12.42 | -4.93 | -4.76 | -4.75 | 0.55 | 0.83 | 0.66 | 1.464085696 | 1.777685362 | 1.580082624 |
| C803O | 16.64 | 16.61 | 16.6 | 12.48 | 12.57 | 12.36 | -4.16 | -4.04 | -4.24 | -1.08 | -1.06 | -1.28 | 0.473028823 | 0.47963206 | 0.411795509 |
| C804K | 16.03 | 15.89 | 15.91 | 14.09 | 13.99 | 13.83 | -1.94 | -1.9 | -2.08 | 1.75 | 1.59 | 1.49 | 3.363585661 | 3.010493495 | 2.808889751 |

## Table S7 The qPCR data of taxifolinproduction strains Y732

| **Strains** | **CT,ACT1** | | | **CT,aroL** | | | **ΔCT** | | | **-ΔΔCT** | | | **Fold Change** | | |
| --- | --- | --- | --- | --- | --- | --- | --- | --- | --- | --- | --- | --- | --- | --- | --- |
| Y200 | 16.94 | 16.78 | 16.82 | 16.52 | 16.51 | 16.55 | -0.42 | -0.27 | -0.27 |  |  |  |  |  |  |
| Y732 | 16.7 | 16.69 | 16.96 | 15.69 | 15.82 | 15.72 | -1.01 | -0.87 | -1.24 | 0.59 | 0.6 | 0.97 | 1.505246747 | 1.515716567 | 1.958840595 |
|  | **CT,ACT1** | | | **ΔCT** | | | **ΔCT** | | | **-ΔΔCT** | | | **Fold Change** | | |
| Y200 | 16.94 | 17 | 16.98 | 16.69 | 16.57 | 16.64 | -0.25 | -0.43 | -0.34 |  |  |  |  |  |  |
| Y732 | 17.49 | 17.43 | 17.44 | 15.35 | 15.39 | 15.23 | -2.14 | -2.04 | -2.21 | 1.89 | 1.61 | 1.87 | 3.706352248 | 3.052518418 | 3.655325801 |
|  | **CT,ACT1** | | | **CT,F3'H** | | | **ΔCT** | | | **-ΔΔCT** | | | **Fold Change** | | |
| C100 | 16.54 | 16.57 | 16.61 | 28.31 | 27.8 | 27.97 | 11.77 | 11.23 | 11.36 |  |  |  |  |  |  |
| Y732 | 17.59 | 17.69 | 17.62 | 25.28 | 25.27 | 24.76 | 7.69 | 7.58 | 7.14 | 4.08 | 3.65 | 4.22 | 16.91228865 | 12.55334557 | 18.63573738 |

## Table S8 The qPCR data of taxifolinproduction strains C901

| **Strains** | **CT,ACT1** | | | **CT,aroL** | | | **ΔCT** | | | **-ΔΔCT** | | | **Fold Change** | | |
| --- | --- | --- | --- | --- | --- | --- | --- | --- | --- | --- | --- | --- | --- | --- | --- |
| Y200 | 14.36 | 14.32 | 14.04 | 12.73 | 12.76 | 12.89 | -1.63 | -1.56 | -1.15 |  |  |  |  |  |  |
| C901 | 16.7 | 16.85 | 16.21 | 12.12 | 12.31 | 12.09 | -4.58 | -4.54 | -4.12 | 2.95 | 2.98 | 2.97 | 7.727490631 | 7.889861636 | 7.835362381 |
|  | **ACT1** | | | **CHS** | | | **ΔCT** | | | **-ΔΔCT** | | | **Fold Change** | | |
| Y200 | 15.96 | 15.97 | 15.77 | 14.58 | 15.36 | 15.25 | -1.38 | -0.61 | -0.52 |  |  |  |  |  |  |
| C901 | 17.48 | 16.51 | 16.89 | 13.87 | 13.57 | 13.99 | -3.61 | -2.94 | -2.9 | 2.23 | 2.33 | 2.38 | 4.691339797 | 5.028053498 | 5.205367422 |
|  | **ACT1** | | | **F3'H** | | | **ΔCT** | | | **-ΔΔCT** | | | **Fold Change** | | |
| C100 | 14.36 | 14.32 | 14.04 | 13.26 | 13.77 | 13.68 | -1.1 | -0.55 | -0.36 |  |  |  |  |  |  |
| C901 | 17.35 | 16.91 | 16.28 | 13.46 | 13.58 | 13.11 | -3.89 | -3.33 | -3.17 | 2.79 | 2.78 | 2.81 | 6.91629785 | 6.868523492 | 7.012845771 |

# REFERENCES

[1] Gao, S.; Zhou, H. R.; Zhou, J. W.; Chen, J., Promoter-library-based pathway optimization for efficient (2*S*)-naringenin production from *p*-coumaric acid in *Saccharomyces cerevisiae*. J Agric Food Chem. 2020, 68 (25), 6884-6891. <https://doi.org/10.1021/acs.jafc.0c01130>

[2] Gao, S.; Lyu, Y. B.; Zeng, W. Z.; Du, G. C.; Zhou, J. W.; Chen, J., Efficient biosynthesis of (2*S*)-naringenin from *p*-coumaric acid in *Saccharomyces cerevisiae*. J Agric Food Chem. 2020, 68 (4), 1015-1021. <https://doi.org/10.1021/acs.jafc.9b05218>

[3] Lv, Y. K.; Xu, S.; Lyu, Y. B.; Zhou, S. H.; Du, G. C.; Chen, J.; Zhou, J. W., Engineering enzymatic cascades for the efficient biotransformation of eugenol and taxifolin to silybin and isosilybin. Green Chem. 2019, 21 (7), 1660-1667. <https://doi.org/10.1039/c8gc03728k>

[4] Gao, S.; Zhou, J.; Chen, J., Identification of flavonoids 3-hydroxylase from [*Silybum marianum* (L.) Gaertn] and its application in enhanced production of taxifolin. Chin J Biotech. 2020, 36 (12), 2838-2849. <https://doi.org/10.13345/j.cjb.200178>

[5] Gao, S.; Xu, X.; Zeng, W.; Xu, S.; Lyv, Y.; Feng, Y.; Kai, G.; Zhou, J.; Chen, J., Efficient biosynthesis of (2*S*)-eriodictyol from (2*S*)-naringenin in *Saccharomyces cerevisiae* through a combination of promoter adjustment and directed evolution. ACS Synth Biol. 2020, 9 (12), 3288-3297. <https://doi.org/10.1021/acssynbio.0c00346>

[6] Maury, J.; Germann, S. M.; Jacobsen, S. A. B.; Jensen, N. B.; Kildegaard, K. R.; Herrgard, M. J.; Schneider, K.; Koza, A.; Forster, J.; Nielsen, J.; Borodina, I., EasyCloneMulti: A set of vectors for simultaneous and multiple genomic integrations in *Saccharomyces cerevisiae*. PloS One. 2016, 11 (3), e0150394. <https://doi.org/10.1371/journal.pone.0150394>

[7] Gueldener, U.; Heinisch, J.; Koehler, G. J.; Voss, D.; Hegemann, J. H., A second set of *loxP* marker cassettes for Cre-mediated multiple gene knockouts in budding yeast. Nucleic Acids Res. 2002, 30 (6), e23. <https://doi.org/10.1093/nar/30.6.e23>

[8] Goldstein, A. L.; McCusker, J. H., Three new dominant drug resistance cassettes for gene disruption in *Saccharomyces cerevisiae*. Yeast. 1999, 15 (14), 1541-1553. <https://doi.org/10.1002/(SICI)1097-0061(199910)15:14><1541::AID-YEA476>3.0.CO;2-K

[9] Entian, K. D.; Kotter, P., Yeast genetic strain and plasmid collections. Method Microbiol. 2007, 36, 629-666. <https://doi.org/10.1016/S0580-9517(06)36025-4>
